# Efficacy of acute care pathways for older patients: a systematic review and meta-analysis

**DOI:** 10.1007/s10433-022-00743-w

**Published:** 2022-11-17

**Authors:** Abtin Ijadi Maghsoodi, Valery Pavlov, Paul Rouse, Cameron G. Walker, Matthew Parsons

**Affiliations:** 1 Department of Information Systems and Operations Management, Faculty of Business and Economics, University of Auckland, Auckland, New Zealand; 2 Department of Intelligence & Insights, Te Whatu Ora Health New Zealand Waikato District, Hamilton, New Zealand; 3 Department of Accounting and Finance, Faculty of Business and Economics , University of Auckland, Auckland, New Zealand; 4 Department of Engineering Science, Faculty of Engineering , University of Auckland, Auckland, New Zealand; 5 School of Health , University of Waikato, Hamilton, New Zealand; 6 Te Whatu Ora Health New Zealand Waikato District, Hamilton, New Zealand

**Keywords:** Acute geriatric pathways, Acute care for older persons, Meta-analysis, Systematic literature review, Randomized controlled trial

## Abstract

Meeting the needs of acute geriatric patients is often challenging, and although evidence shows that older patients need tailored care, it is still unclear which interventions are most appropriate. The objective of this study is to systematically evaluate the hospital-wide acute geriatric models compared with conventional pathways. The design of the study includes hospital-wide geriatric-specific models characterized by components including patient-centered care, frequent medical review, early rehabilitation, early discharge planning, prepared environment, and follow‐up after discharge. Primary and secondary outcomes were considered, including functional decline, activities of daily living (ADL), length-of-stay (LoS), discharge destination, mortality, costs, and readmission. A systematic review and meta-analysis were conducted in accordance with the Preferred Reporting Items for Systematic Reviews and Meta-Analysis (PRISMA) guidelines. A total of 20 studies reporting on 15 trials and acutely admitted patients with an average age of 79, complex conditions and comorbidities to acute geriatric-specific pathways (*N* = 13,595) were included. Geriatric-specific models were associated with lower costs (weighted mean difference, WMD =  − $174.98, 95% CI = -$332.14 to − $17.82; *P* = 0.03), and shorter LoS (WMD =  − 1.11, 95% CI =  − 1.39 to − 0.83; *P* < 0.001). No differences were found in functional decline, ADL, mortality, case fatalities, discharge destination, or readmissions. Geriatric-specific models are valuable for improving patient and system-level outcomes. Although several interventions had positive results, further research is recommended to study hospital-wide geriatric-specific models.

**Supplementary Information:**

The online version contains supplementary material available at 10.1007/s10433-022-00743-w.

## Introduction

A significant accomplishment of the current century is the gain of approximately 30 years in life expectancy (Christensen et al. 2009). However, longevity presents challenges including but not limited to shifting disease burden, increased expenditure on health and long-term care, labor-force shortages, dissaving, functioning and quality of life, and potential problems with old-age income security. In addition, older people (65 + years) account for the majority of hospitalized patients in acute care settings (Steele 2010), and while in hospital, they experience a higher rate of adverse outcomes, including all-cause in-hospital mortality and a longer length of hospital stay associated with various events including but not limited to polypharmacy, sarcopenia, pressure ulcer, health care acquired pneumonia and other iatrogenic problems, gastrointestinal bleeding, and more life-threatening events such as sepsis and opioid overdose (Palmer 2018). Hence, optimizing the journey of an older person through an acute hospital has become a significant policy and clinical imperative (Hickman et al. 2015). Over the past three decades, various tailored geriatric-specific models have been developed with a focus on improving health outcomes, level of independence, functioning, and quality of life (Boockvar et al. 2020; Landefeld 2003; Rubenstein et al. 1984; Sanon et al. 2019). Examples of popular older person-specific models are Acute Care for Elders—ACE (Landefeld et al. 1995), Hospitalized Elder Life Program—Help (Inouye et al. 1999), and Geriatric Evaluation and Management Units—GEMU (Ellis & Langhorne 2004).

The most popular older person-specific model is the ACE program, applied to different wards, including emergency departments (Sanon et al. 2019), and inpatient units (Palmer 2018). Several papers have systematically reviewed geriatric-specific units in various settings (Bakker et al. 2011; Baztan et al. 2009; Fox et al. 2012, 2013; Van Craen et al. 2010). Fox et al. (2013) presented a systematic review of ACE model components and outcomes considering iatrogenic complexities, functional decline, length of stay (LoS), costs, and discharge to home. Baztan et al. (2009) analyzed randomized trials, non-randomized trials, and case–control studies to assess the effectiveness of acute geriatric units compared with conventional units. Although previous systematic literature reviews explored the components of geriatric-specific units, with a focus on specific models of care (Baztan et al. 2009; Fox et al. 2012; Hickman et al. 2015), they have not provided a meta-analysis of randomized controlled trials (RCTs) for the patient journey from admission to discharge, based on hospital-wide interventions. Bakker et al. (2011) coined the term "hospital-wide" models of care as integrated system interventions, not restricted to medical specialities, units or departments, that are available for all older hospitalized patients.

This research conducts a systematic literature review and meta-analysis of geriatric-specific RCTs in hospital-wide interventions. Since the risk of poor functional outcomes can occur at any point in the patients' journey, the entire pathway must be analyzed. The primary aim of this review is to analyze findings and determine the principal components of RCTs of acute older person-specific interventions during the acute phase of a patient's illness tailored to improve clinical and operational outcomes.

## Methods

A systematic review and meta-analysis was performed comparing the outcomes of care in hospital-wide acute older person-specific services with conventional pathways in accordance with the Preferred Reporting Items for Systematic Reviews and Meta-Analysis (PRISMA) guidelines (Liberati et al. 2009; Shamseer et al. 2015), and the Cochrane Handbook for Systematic Reviews of Interventions (Cumpston et al. 2019). This systematic review was registered on PROSPERO with registration number CRD42021224860.

### Eligibility criteria

Eligible studies included RCTs that compared acute older person-specific models with conventional care. Participants of the studies were patients aged 65 + who were acutely admitted to hospital care, including unplanned, unscheduled, or acute presentations, which may have had comorbidities or iatrogenic conditions. The search was limited to publications in the English language from 1995 to 2021, as it was believed that older results would no longer apply due to recent developments in medicine and healthcare. Eligible studies had at least one component of older person-specific interventions, and geriatric-specific models of care in a hospital-wide setting were defined as any additional package of care designed explicitly for a geriatric patient to improve health outcomes at any point in the patients' journey throughout the hospital system, which are not limited to any medical departments, units, or specialties. An acute older person model of care was defined with at least one of the following components at any point of patient's journey (Baztan et al. 2009; Fox et al. 2012; Palmer 2018): patient-centered care^1^ as defined by Fox et al. (2012) in their respective review; activities to prevent declines in ADL, mobility, continence, nutrition, and cognition with a focus on interdisciplinary team-based care, and specific restorative guidelines on patient mobility, ADL functioning, patient nutrition with specific goals, and attention to skin integrity, urinary and bowel continence, cognitive function (including maintaining/restoring normal wake and sleep cycles, and prevention of delirium), and augmenting hearing and vision; frequent medical review; activities to minimize the adverse effects of treatments on older adults' functioning; early rehabilitation; the participation of physical/occupational therapists to initiate rehabilitation or provision of physical/occupational therapy; early discharge planning; activities to facilitate return to the community; and prepared environment; environmental modifications to facilitate physical and cognitive functioning. Usual care was defined as any care not provided on an acute geriatric pathway.

Eligible studies comprised at least one of the following primary outcomes: functional decline, comorbidities and iatrogenic complications. In which, comorbidities included chronic conditions such as hypertension, hyperlipidemia, ischemic heart disease, diabetes, arthritis, heart failure, depression, chronic kidney disease, osteoporosis, Alzheimer's disease, chronic obstructive pulmonary disease, atrial fibrillation, cancer, asthma, stroke (patients who experienced ≥ 1 coexisting conditions) (Gontijo Guerra et al. 2019). Iatrogenic complications investigated in the current analysis are: falls, pressure ulcers, delirium, adverse drug reactions, and anaphylaxis (patients who experienced ≥ 1 of such conditions) (Krishnan and Kasthuri 2005). Additionally, the secondary outcomes investigated were: length of stay (LoS), destination after discharge (home, institutional or rehabilitation care), mortality, costs, and readmission within a minimum of 30 days following discharge. In which, costs were defined as total costs and charges ($US) for the duration of the stay (Baztan et al. 2009; Fox et al. 2012).

The exclusion criteria comprised studies that were not peer-reviewed and studies that did not have an age threshold of 65 + years. Elective admissions for surgical procedures were excluded. Studies of organized care for specific conditions, including stroke units, orthopedic units, surgical units, medical-surgical units, and surgical admissions, were excluded. A brief summary and overview of the eligibility criteria are provided in Table 1.

**Table 1:** Eligibility criteria of the study

Inclusion criteria	Exclusion criteria
Randomized Control Trials	Non-Randomized Control Trials
Publications in the English language	Publications in other languages
Studies published from 1995 to 2021	Studies published before 1995
Peer-reviewed studies	Patients aged 65 years
Patients aged 65 + years	Elective admissions for surgical procedures
Studies comparing acute older person-specific models with conventional care	Studies of organized care for specific conditions, including stroke units, orthopedic units, surgical units, medical–surgical units, and surgical admissions
Acute unplanned and unscheduled admissions with comorbidities or iatrogenic conditions At least one component of geriatric-specific interventions in a hospital-wide setting including patient-centered care, frequent medical review, early rehabilitation, early discharge planning At least one of the primary outcomes such as functional decline, comorbidities and iatrogenic complications, and secondary outcomes investigated including: length of stay (LoS), destination after discharge (home, institutional or rehabilitation care), mortality, costs, and readmission within a minimum of 30 days following discharge	

### Search strategy and study selection

The literature search was led by A.I.M assisted by a subject librarian to pinpoint medical subject headings and keywords reflective of the inclusion criteria. Electronic databases searched were EMBASE, PubMed, CINAHL, OECD health policies and data, NHSEED, Web of Science, SCOPUS, SSRN, Cochrane Library, and ProQuest. Hand searching was conducted in publisher databases, including ScienceDirect, SpringerLink, Sage, and Wiley. A manual hand search and text word search was also conducted within the abstracts and titles of the mentioned databases and in high-impact journals in the field of gerontology, including the Journal of the American Geriatrics Society (JAGS), Archives of Gerontology and Geriatrics, Age and Ageing, Journal of the American Medical Association, Gerontologist, Journals of Gerontology, and bibliography information of comprised studies and previous reviews aligned with the inclusion criteria. The search strategy using the medical subject headings and other extracted keywords is presented in supplementary Appendix S1. The last update for the current review was performed on October 5, 2021. Titles and abstracts were reviewed in the first screening stage, and articles not meeting the inclusion criteria were excluded. Full-text papers were retrieved for further review, and in cases of insufficient information in the title and abstract, the complete article was reviewed. It should be noted that for the eligible and included research studies forward citation search was also included. Three reviewers (A.I.M, P.R., and M.P) were involved with the eligibility assessment of the studies. Two reviewers (A.I.M and M.P) independently assessed the eligibility of studies retrieved from the literature search for potential inclusion. In cases where consensus could not be achieved, a third team member (P.R) reviewed the studies to reach a concluding consensus.

### Data extraction and risk of bias assessment

The quality assessment of the eligible papers was conducted independently by two reviewers (A.I.M and M.P). Discrepancies were resolved through discussion with a third reviewer (P.R). A total of 20 study authors were contacted where additional data were required. Information categories extracted for the review included design, setting, time of assessment, participants, comparison groups of intervention, and conventional care containing information such as primary diagnostic symptom and category, iatrogenic complexities, comorbidities, and critical elements. Risk of bias assessment was conducted independently by two reviewers (A.I.M and M.P) using the Effective Practice and Organization of Care (EPOC) checklist, including the following criteria: random sequence generation, allocation concealment, similar baseline outcome measures, similar baseline characteristics, incomplete outcome data addressed, prevention knowledge allocated interventions, adequate protection against contamination, free of selective reporting, and free of other bias (Cumpston et al. 2019). The overall quality was also summarized with the Jadad scale (Baztan et al. 2009; Jadad et al. 1996). Jadad Scale consists of three items, including randomization (maximum of 2 points), blinding (maximum of 2 points)—excluded, and account of all patients (maximum of 1 point). For some studies, it was not possible for acute geriatric units to exclude the blinding of the intervention, this scale has been used for that type of study (Jadad et al. 1996).

### Data analysis and synthesis

Data regarding characteristics, primary and secondary measures compared to conventional care were extracted and synthesized to describe the impact of the interventions on different outcomes in patients' journeys. The meta-analysis was of RCT studies with a low risk of bias. The statistical analysis was conducted using the Cochrane Review Manager software (Cochrane RevMan 5.4.1, Cochrane Collaboration, Oxford, UK) (Cumpston et al. 2019). Continuous and dichotomous outcomes were examined using a random-effects model to calculate weighted mean differences (WMD) and risk ratios (RR), with a confidence interval (CI) of 95% (Cumpston et al. 2019). While $P<0.05$ was considered statistically significant for an overall effect, $P<0.10$ was considered significant for heterogeneity (Fox et al. 2012). Heterogeneity was quantified using the ${I}^{2}$ statistic and was considered significant when ${I}^{2}$ was more than 40%. Where it was possible to combine the results, a random-effects method was utilized (Baztan et al. 2009; Higgins et al. 2003). Due to the potential for heterogeneity of populations and intervention components, there is a possibility of having a false-negative ${I}^{2}$ statistic. Therefore, CIs of individual studies included in the forest plots were also examined. A sensitivity analysis was performed to determine the robustness of findings where heterogeneity was statistically significant. Decisions for removing studies from the meta-analysis and synthesis were based on the risk of bias and possible sources of variability. Based on the data extracted from the studies and possible sources of variability, studies that did not contain similar components of an older person-specific model were removed, starting with studies that contained the fewest components.

## Results

### Description and characteristics of studies

**Fig. 1:**
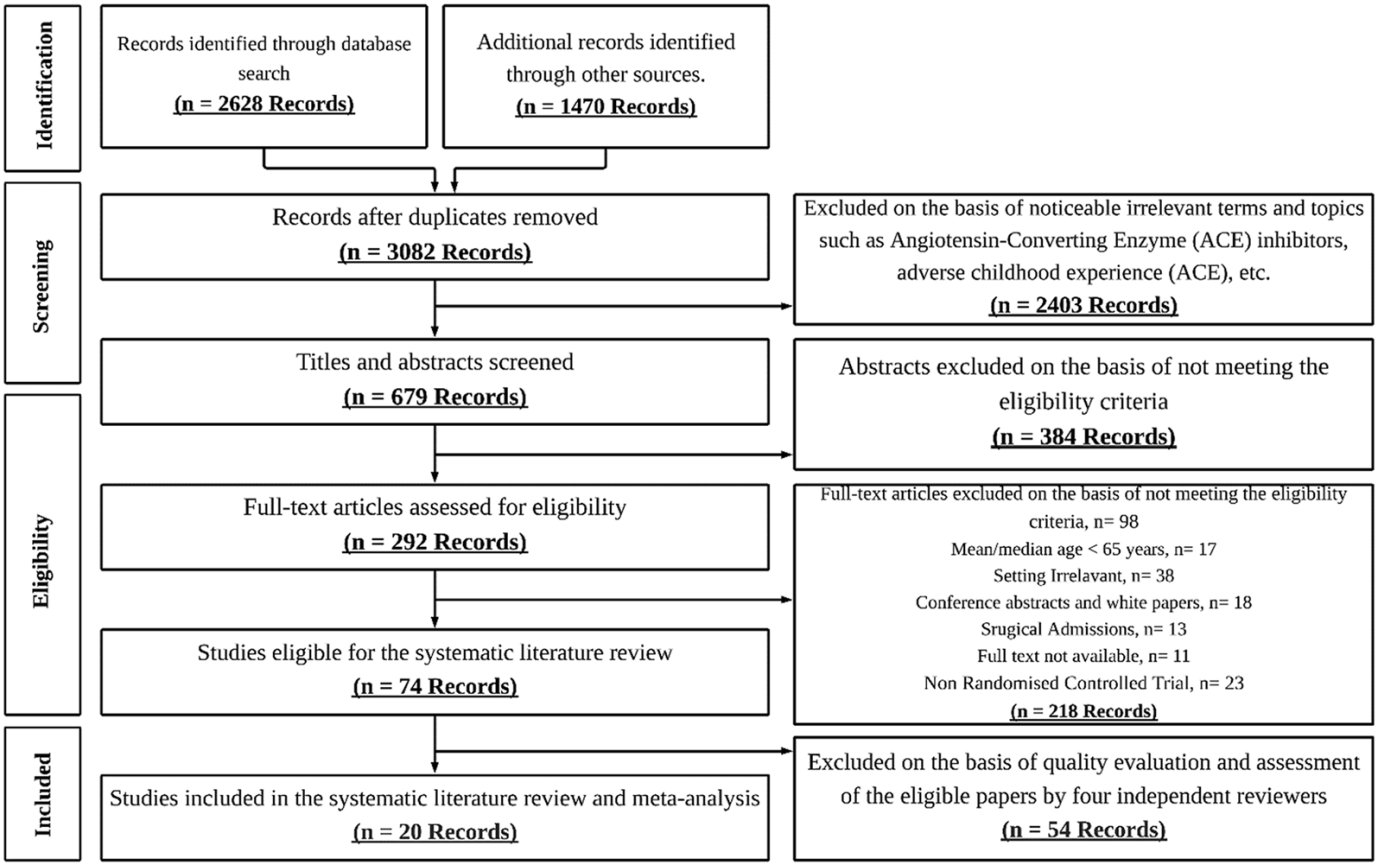
PRISMA flow diagram for selecting studies

The literature search of all sources yielded 3082 unique citations, of which 74 were selected for critical and comprehensive reading after reviewing the titles and abstracts. A total of 20 studies reporting on 15 trials met the inclusion criteria (Asplund et al. 2000; Barnes et al. 2012 Buurman et al. 2016; Cohen et al. 2002; Coleman et al. 2006; Counsell et al. 2000; Covinsky et al. 1997; Kircher et al. 2007; Landefeld et al. 1995; Legrain et al. 2011; McCusker et al. 2001; Phibbs et al. 2006; Reuben et al. 1995; Saltvedt et al. 2002, 2004, 2005, 2006; Tibaldi et al. 2009; Wald et al. 2011; Westgard et al. 2020) (Fig. 1). Nine studies were conducted in the USA (Barnes et al. 2012; Cohen et al. 2002; Coleman et al. 2006; Counsell et al. 2000; Covinsky et al. 1997; Landefeld et al. 1995; Phibbs et al. 2006; Reuben et al. 1995; Wald et al. 2011), two in Sweden (Asplund et al. 2000; Westgard et al. 2020), four in Norway (Saltvedt et al. 2002, 2004, 2005, 2006), and the remainder in Canada (McCusker et al. 2001), Germany (Kircher et al. 2007), Italy (Tibaldi et al. 2009), France (Legrain et al. 2011), and Netherlands (Buurman et al. 2016).

In total, the included studies comprised 13,595 participants, of which 51.48% were in the intervention group. The average participant was aged 79, female (54%), and admitted with at least one principal diagnosis (75%) (Asplund et al. 2000; Barnes et al. 2012; Buurman et al. 2016; Coleman et al. 2006; Counsell et al. 2000; Covinsky et al. 1997; Landefeld et al. 1995; Legrain et al. 2011; McCusker et al. 2001; Saltvedt et al. 2002, 2004, 2005, 2006; Tibaldi et al. 2009; Wald et al. 2011), or other morbidities (25%) (Cohen et al. 2002; Kircher et al. 2007; Phibbs et al. 2006; Reuben et al. 1995; Westgard et al. 2020). In 55% of the studies, patients came exclusively from medical or general wards (Barnes et al. 2012; Buurman et al. 2016; Cohen et al. 2002; Counsell et al. 2000; Kircher et al. 2007; Phibbs et al. 2006; Saltvedt et al. 2002, 2004, 2005, 2006; Wald et al. 2011), and in 35% of studies, patients were admitted through an emergency department (Asplund et al. 2000; Covinsky et al. 1997; Landefeld et al. 1995; Legrain et al. 2011; McCusker et al. 2001; Tibaldi et al. 2009; Westgard et al. 2020). A total of 10% of patients were admitted through other departments or did not provide this information (Coleman et al. 2006; Reuben et al. 1995). While descriptive characteristics of the 20 studies are provided in Supplementary Table S1 with details, a very short summary of the study characteristics just including age, gender, and country of the study is presented in Table 2.

**Table 2:** Brief summary of the characteristics of studies included in the systematic literature review

Variables	Number of patients (%)	
	Intervention (*n* = 7000)	Control (*n* = 6595)
Age (mean years ± standard deviation)	79.385 ± 7.4	79.325 ± 6.7
Gender		
Male	3203 (44.24)	3044 (40.76)
Female	3797 (55.77)	3551 (56.38)
Country		
USA	5158 (73.68)	4710 (71.41)
Sweden	268 (3.82)	300 (4.54)
Norway	508 (7.25)	508 (7.70)
France	317 (4.52)	348 (5.27)
Netherlands	337 (4.81)	337 (5.10)
Canada	178 (2.54)	210 (3.18)
Germany	186 (2.65)	129 (1.95)
Italy	48 (0.68)	53 (0.80)

Acute older person care models consisted of patient-centered care (Barnes et al. 2012; Cohen et al. 2002; Coleman et al. 2006; Counsell et al. 2000; Covinsky et al. 1997; Kircher et al. 2007; Landefeld et al. 1995; Legrain et al. 2011; McCusker et al. 2001; Phibbs et al. 2006; Reuben et al. 1995; Saltvedt et al. 2002, 2004, 2005, 2006; Westgard et al. 2020), frequent medical review (Barnes et al. 2012; Coleman et al. 2006; Counsell et al. 2000; Covinsky et al. 1997; Landefeld et al. 1995; Legrain et al. 2011; Reuben et al. 1995; Tibaldi et al. 2009; Wald et al. 2011; Westgard et al. 2020), early rehabilitation (Asplund et al. 2000; Barnes et al. 2012; Cohen et al. 2002; Counsell et al. 2000; Covinsky et al. 1997; Kircher et al. 2007; Landefeld et al. 1995; Phibbs et al. 2006; Saltvedt et al. 2002, 2004, 2005, 2006; Tibaldi et al. 2009), early discharge planning (Asplund et al. 2000; Barnes et al. 2012; Cohen et al. 2002; Counsell et al. 2000; Covinsky et al. 1997; Kircher et al. 2007; Landefeld et al. 1995; Legrain et al. 2011; Phibbs et al. 2006;Saltvedt et al. 2002, 2004, 2005, 2006; Tibaldi et al. 2009; Wald et al. 2011), and prepared environment (Barnes et al. 2012; Counsell et al. 2000; Covinsky et al. 1997; Landefeld et al. 1995; Westgard et al. 2020). Core teams were designed predominantly with a team of physicians and registered nurses (Asplund et al. 2000; Barnes et al. 2012; Buurman et al. 2016; Cohen et al. 2002; Coleman et al. 2006; Counsell et al. 2000; Covinsky et al. 1997; Kircher et al. 2007; Landefeld et al. 1995; Legrain et al. 2011; McCusker et al. 2001; Phibbs et al. 2006; Reuben et al. 1995; Saltvedt et al. 2002, 2004, 2005, 2006; Tibaldi et al. 2009; Wald et al. 2011; Westgard et al. 2020), geriatricians (Asplund et al. 2000; Barnes et al. 2012; Buurman et al. 2016; Cohen et al. 2002; Counsell et al. 2000; Covinsky et al. 1997; Kircher et al. 2007; Landefeld et al. 1995; Legrain et al. 2011; McCusker et al. 2001; Reuben et al. 1995; Saltvedt et al. 2002, 2004, 2005, 2006; Tibaldi et al. 2009; Wald et al. 2011; Westgard et al. 2020), physiotherapists (Asplund et al. 2000; Barnes et al. 2012; Counsell et al. 2000; Covinsky et al. 1997; Kircher et al. 2007; Landefeld et al. 1995; Saltvedt et al. 2002, 2004, 2005, 2006; Tibaldi et al. 2009; Wald et al. 2011), nutritionists (Barnes et al. 2012; Covinsky et al. 1997; Landefeld et al. 1995; Wald et al. 2011; Westgard et al. 2020), social workers (Asplund et al. 2000; Barnes et al. 2012; Cohen et al. 2002; Counsell et al. 2000; Covinsky et al. 1997; Kircher et al. 2007; Landefeld et al. 1995; Reuben et al. 1995; Tibaldi et al. 2009; Wald et al. 2011; Westgard et al. 2020), and occupational therapists (Asplund et al. 2000; Saltvedt et al. 2002, 2004, 2005, 2006; Westgard et al. 2020). Interdisciplinary teams met regularly to plan patient care (Asplund et al. 2000; Barnes et al. 2012; Buurman et al. 2016; Cohen et al. 2002; Coleman et al. 2006; Counsell et al. 2000; Covinsky et al. 1997; Kircher et al. 2007; Landefeld et al. 1995; Legrain et al. 2011; McCusker et al. 2001; Phibbs et al. 2006; Reuben et al. 1995; Saltvedt et al. 2006, 2002, 2004, 2005; Tibaldi et al. 2009; Wald et al. 2011; Westgard et al. 2020). The follow-up period in studies varied from 1 to 12 months (Asplund et al. 2000; Buurman et al. 2016; Cohen et al. 2002; Coleman et al. 2006; Counsell et al. 2000; Covinsky et al. 1997; Kircher et al. 2007; Landefeld et al. 1995; Legrain et al. 2011; McCusker et al. 2001; Phibbs et al. 2006 Reuben et al. 1995 Saltvedt et al. 2002, 2004, 2005, 2006; Tibaldi et al. 2009, Westgard et al. 2020), except two studies (Barnes et al. 2012; Wald et al. 2011) which did not report follow-ups. Patients assigned to conventional care were eligible to receive all standard treatments ordered by their primary nurses, physicians, and services in eleven studies (Asplund et al. 2000; Barnes et al. 2012; Cohen et al. 2002; Coleman et al. 2006; Kircher et al. 2007; McCusker et al. 2001; Phibbs et al. 2006; Reuben et al. 1995; Tibaldi et al. 2009; Wald et al. 2011; Westgard et al. 2020).

### Risk of bias in studies

The risk of selection bias resulting from random sequence generation was low in nineteen studies (Asplund et al. 2000; Barnes et al. 2012; Buurman et al. 2016; Cohen et al. 2002; Coleman et al. 2006; Counsell et al. 2000; Covinsky et al. 1997; Kircher et al. 2007; Landefeld et al. 1995; Legrain et al. 2011; McCusker et al. 2001; Phibbs et al. 2006; Reuben et al. 1995; Saltvedt et al. 2002, 2004, 2005, 2006; Tibaldi et al. 2009, Westgard et al. 2020). One study (Wald et al. 2011) had high risk of random sequence generation due to conducting a Quasi-RCT. Risk of selection bias resulting from allocation concealment was low in seventeen studies (Asplund et al. 2000; Barnes et al. 2012; Buurman et al. 2016; Cohen et al. 2002; Coleman et al. 2006; Counsell et al. 2000; Covinsky et al. 1997; Landefeld et al. 1995; Legrain et al. 2011; McCusker et al. 2001; Phibbs et al. 2006; Saltvedt et al. 2002, 2004, 2005, 2006; Tibaldi et al. 2009; Westgard et al. 2020). Three studies (Kircher et al. 2007; Reuben et al. 1995; Wald et al. 2011), that used randomization provided insufficient information to draw conclusions in this domain. Possible risk of adequate protection against contamination was low in nine studies (Buurman et al. 2016; Coleman et al. 2006; McCusker et al. 2001; Saltvedt et al. 2002, 2004, 2005, 2006; Tibaldi et al. 2009, Westgard et al. 2020). Nine studies were unable to eliminate the possibility of contamination, resulting in a high-risk assessment (Asplund et al. 2000; Barnes et al. 2012; Cohen et al. 2002; Counsell et al. 2000; Covinsky et al. 1997; Kircher et al. 2007; Landefeld et al. 1995; Phibbs et al. 2006; Reuben et al. 1995). In all other studies, risk of bias was unclear because information about protection against contamination was not provided (Legrain et al. 2011; Wald et al. 2011). Possible risk of similar baseline outcome measures was low (Asplund et al. 2000; Barnes et al. 2012; Buurman et al. 2016; Cohen et al. 2002; Counsell et al. 2000; Covinsky et al. 1997; Kircher et al. 2007; Legrain et al. 2011; McCusker et al. 2001; Phibbs et al. 2006; Reuben et al. 1995; Saltvedt et al. 2002, 2004, 2005, 2006; Tibaldi et al. 2009; Westgard et al. 2020), or unclear (Coleman et al. 2006; Wald et al. 2011). One study (Landefeld et al. 1995) had a high risk of similar baseline outcome measures because of its lack of adjustment in analysis. While the possible risk of bias due to relevant differences in baseline characteristics was low in sixteen studies (Asplund et al. 2000; Buurman et al. 2016; Cohen et al. 2002; Coleman et al. 2006; Covinsky et al. 1997; Kircher et al. 2007; Landefeld et al. 1995; Legrain et al. 2011; McCusker et al. 2001; Phibbs et al. 2006; Reuben et al. 1995; Saltvedt et al. 2002, 2004, 2005, 2006; Tibaldi et al. 2009), four studies were considered to have a high risk (Barnes et al. 2012; Counsell et al. 2000; Wald et al. 2011; Westgard et al. 2020). Knowledge of the allocated interventions was adequately prevented during six studies (Buurman et al. 2016; Cohen et al. 2002; Covinsky et al. 1997; Kircher et al. 2007; Phibbs et al. 2006; Tibaldi et al. 2009), and unclear in nine studies (Legrain et al. 2011; McCusker et al. 2001; Reuben et al. 1995; Saltvedt et al. 2002, 2004, 2005, 2006; Wald et al. 2011, Westgard et al. 2020). Outcomes were not assessed blindly in five studies (Asplund et al. 2000; Barnes et al. 2012; Coleman et al. 2006; Counsell et al. 2000; Landefeld et al. 1995). The possible risk of incomplete outcome data was low in seven studies (Barnes et al. 2012; Buurman et al. 2016; Counsell et al. 2000; Kircher et al. 2007; Legrain et al. 2011; Tibaldi et al. 2009; Wald et al. 2011), or unclear in 13 studies (Asplund et al. 2000; Cohen et al. 2002; Coleman et al. 2006; Covinsky et al. 1997; Landefeld et al. 1995; McCusker et al. 2001; Phibbs et al. 2006; Reuben et al. 1995; Saltvedt et al. 2002, 2004, 2005, 2006; Westgard et al. 2020). Risk of reporting bias due to selective reporting was low (16 studies) (Barnes et al. 2012; Buurman et al. 2016; Cohen et al. 2002; Coleman et al. 2006; Counsell et al. 2000; Covinsky et al. 1997; Kircher et al. 2007; Legrain et al. 2011; Phibbs et al. 2006; Reuben et al. 1995; Saltvedt et al. 2002, 2004, 2005, 2006; Tibaldi et al. 2009, Wald et al. 2011), or unclear (Asplund et al. 2000; Landefeld et al. 1995; McCusker et al. 2001; Westgard et al. 2020). None of the twenty studies appeared to have the risk of other sources of bias that were not addressed in the prior assessment areas. A summary of the potential sources of bias assessment is presented in Table 3.

**Table 3:** Summary of the potential sources of bias assessment

Study Author Info (Year)	Random sequence generation	Allocation concealment	Similar baseline outcome measures	Similar baseline characteristics	Incomplete outcome data addressed	Prevention knowledge allocated interventions	Adequate protection against contamination	Free of selective reporting	Free of Other Bias	Jadad Scale §
Landefeld et al. (1995)	( +)	( +)	(−)	( +)	(?)	(−)	(−)	(?)	( +)	3
Reuben et al. (1995)	( +)	(?)	( +)	( +)	(?)	(?)	(−)	( +)	( +)	2
Covinsky et al. (1997)	( +)	( +)	( +)	( +)	(?)	( +)	(−)	( +)	( +)	3
Counsell et al. (2000)	( +)	( +)	( +)	(−)	( +)	(−)	(−)	( +)	( +)	3
Asplund et al. (2000)	( +)	( +)	( +)	( +)	(?)	(−)	(−)	(?)	( +)	2
McCusker et al. (2001)	( +)	( +)	( +)	( +)	(?)	(?)	( +)	(?)	( +)	2
Cohen et al. (2002)	( +)	( +)	( +)	( +)	(?)	( +)	(−)	( +)	( +)	3
Saltvedt et al. (2002)	( +)	( +)	( +)	( +)	(?)	(?)	( +)	( +)	( +)	2
Saltvedt et al. (2004)	( +)	( +)	( +)	( +)	(?)	(?)	( +)	( +)	( +)	2
Saltvedt et al. (2005)	( +)	( +)	( +)	( +)	(?)	(?)	( +)	( +)	( +)	2
Coleman et al. (2006)	( +)	( +)	(?)	( +)	(?)	(−)	( +)	( +)	( +)	2
Phibbs et al. (2006)	( +)	( +)	( +)	( +)	(?)	( +)	(−)	( +)	( +)	3
Saltvedt et al. (2006)	( +)	( +)	( +)	( +)	(?)	(?)	( +)	( +)	( +)	2
Kircher et al. (2007)	( +)	(?)	( +)	( +)	( +)	( +)	(−)	( +)	( +)	3
Tibaldi et al. (2009)	( +)	( +)	( +)	( +)	( +)	( +)	( +)	( +)	( +)	3
Legrain et al. (2011)	( +)	( +)	( +)	( +)	( +)	(?)	(?)	( +)	( +)	2
Wald et al. (2011)	(−)	(?)	(?)	(−)	( +)	(?)	(?)	( +)	( +)	2
Barnes et al. (2012)	( +)	( +)	( +)	(−)	( +)	(−)	(−)	( +)	( +)	3
Buurman et al. (2016)	( +)	( +)	( +)	( +)	( +)	( +)	( +)	( +)	( +)	3
Boockvar et al. (2020)	( +)	( +)	( +)	(−)	(?)	(?)	( +)	(?)	( +)	2

( +): Low Risk, ( −): High Risk, (?): Unclear Risk/Not Reported. § Jadad Scale consists of three items including Randomization (maximum of 2 points), blinding (maximum of 2 points)—excluded, and account of all patients (maximum of 1 point). Refer to (Jadad et al. 1996) for more information. For some studies, it was not possible for acute geriatric units to exclude the blinding of the intervention, this scale has been used for that type of studies

### Effectiveness of hospital-wide geriatric-specific interventions

Ten meta-analyses were performed based on the available data of the eligible studies. Unpublished data were obtained from study authors to conduct meta-analyses on ADL (Asplund et al. 2000; Barnes et al. 2012; Counsell et al. 2000; Landefeld et al. 1995; Reuben et al. 1995; Saltvedt et al. 2006, 2002, 2004, 2005; Westgard et al. 2020), functional decline (Barnes et al. 2012; Counsell et al. 2000; Landefeld et al. 1995; McCusker et al. 2001; Westgard et al. 2020), mortality (Asplund et al. 2000; Barnes et al. 2012; Buurman et al. 2016; Cohen et al. 2002; Counsell et al. 2000; Covinsky et al. 1997; Kircher et al. 2007; Landefeld et al. 1995; Legrain et al. 2011; Saltvedt et al. 2006, 2002, 2004, 2005; Tibaldi et al. 2009), case fatality at discharge (Asplund et al. 2000; Buurman et al. 2016; Counsell et al. 2000; Covinsky et al. 1997; Landefeld et al. 1995; Tibaldi et al. 2009), follow-up (Asplund et al. 2000; Cohen et al. 2002; Counsell et al. 2000; Covinsky et al. 1997; Kircher et al. 2007; Landefeld et al. 1995; Legrain et al. 2011; Saltvedt et al. 2006, 2002, 2004, 2005; Tibaldi et al. 2009), readmission (Asplund et al. 2000; Barnes et al. 2012; Buurman et al. 2016; Coleman et al. 2006; Counsell et al. 2000; Kircher et al. 2007; Landefeld et al. 1995; Legrain et al. 2011; Reuben et al. 1995; Tibaldi et al. 2009; Wald et al. 2011), costs (Asplund et al. 2000; Barnes et al. 2012; Cohen et al. 2002; Coleman et al. 2006; Counsell et al. 2000; Covinsky et al. 1997; Phibbs et al. 2006; Tibaldi et al. 2009; Wald et al. 2011), Length of Stay (LoS) (Barnes et al. 2012; Cohen et al. 2002; Coleman et al. 2006; Counsell et al. 2000; Covinsky et al. 1997; Phibbs et al. 2006; Saltvedt et al. 2006, 2002, 2004, 2005; Tibaldi et al. 2009; Wald et al. 2011), and living situation at discharge (Asplund et al. 2000; Barnes et al. 2012; Buurman et al. 2016; Counsell et al. 2000; Covinsky et al. 1997; Landefeld et al. 1995; Tibaldi et al. 2009; Wald et al. 2011). Sensitivity analysis was performed for ADL, readmission, LoS, and costs. A summary of the meta-analyses is provided in Table 4. All forest plots are available in the electronic supplementary Appendix S2.

**Table 4:** Summary of the meta-analysis results

Outcome Measure [Studies Included in Meta-analysis]	*N* (Total)	WMD (95% CI) or RR (95% CI) ^a^	Test for Overall Effect, Z ($p$-Value)	${I}^{2}$ Statistic ($p$-Value) for Heterogeneity
Functional Decline (Barnes et al. 2012; Counsell et al. 2000; Landefeld et al. 1995; McCusker et al. 2001; Westgard et al. 2020)	4239	0.94 (0.87, 1.01)	1.61 (0.11)	16% (0.31)
Activity of Daily Living (Asplund et al. 2000; Barnes et al. 2012; Counsell et al. 2000; Landefeld et al. 1995; Reuben et al. 1995; Saltvedt et al. 2002, 2004, 2005, 2006; Westgard et al. 2020)	6362	1.09 (0.97, 1.22)	1.46 (0.15)	63% (0.01)
Outliers Removed	5367	1.02 (0.94, 1.10)	0.43 (0.67)	35% (0.19)
Mortality (Asplund et al. 2000; Barnes et al. 2012; Buurman et al. 2016; Cohen et al. 2002; Counsell et al. 2000; Covinsky et al. 1997; Kircher et al. 2007; Landefeld et al. 1995; Legrain et al. 2011; Saltvedt et al. 2006, 2002, 2004, 2005; Tibaldi et al. 2009)	7496	0.92 (0.79, 1.07)	1.05 (0.29)	23% (0.22)
Case Fatality at Discharge (Asplund et al. 2000; Buurman et al. 2016; Counsell et al. 2000; Covinsky et al. 1997; Landefeld et al. 1995; Tibaldi et al. 2009)	4020	0.87 (0.72, 1.05)	1.46 (0.14)	0% (0.80)
Case Fatality at Follow-up (Asplund et al. 2000; Cohen et al. 2002; Counsell et al. 2000; Covinsky et al. 1997; Kircher et al. 2007; Landefeld et al. 1995; Legrain et al. 2011; Saltvedt et al. 2006, 2002, 2004, 2005; Tibaldi et al. 2009)	6326	0.97 (0.87, 1.08)	0.53 (0.60)	Total: 7% (0.37) Subgroup: 0% (0.76)
Living Situation at Discharge (Asplund et al. 2000; Barnes et al. 2012; Buurman et al. 2016; Counsell et al. 2000; Covinsky et al. 1997; Landefeld et al. 1995; Tibaldi et al. 2009; Wald et al. 2011)	5698	1.06 (1.01, 1.12)	2.25 (0.02)	24% (0.24)
Living Situation at Follow-up (Asplund et al. 2000; Counsell et al. 2000; Covinsky et al. 1997; Kircher et al. 2007; Landefeld et al. 1995; Saltvedt et al. 2006, 2002, 2004, 2005)	4031	1.11 (1.03, 1.20)	2.66 (0.008)	Total: 43% (0.10) Subgroup: 75.5% (0.02)
Readmission (Asplund et al. 2000; Barnes et al. 2012; Buurman et al. 2016; Coleman et al. 2006; Counsell et al. 2000; Kircher et al. 2007; Landefeld et al. 1995; Legrain et al. 2011; Reuben et al. 1995; Tibaldi et al. 2009; Wald et al. 2011)	8239	0.98 (0.88, 1.09)	0.32 (0.75)	49% (0.04)
Outliers removed	5721	0.98 (0.87, 1.11)	0.28 (0.78)	39% (0.11)
Costs ^b^ (Asplund et al. 2000; Barnes et al. 2012; Cohen et al. 2002; Coleman et al. 2006; Counsell et al. 2000; Covinsky et al. 1997; Phibbs et al. 2006; Tibaldi et al. 2009; Wald et al. 2011)	6680	−401.13 (−821.43, 19.17)	1.87 (0.06)	84% (< 0.001)
Outliers removed	5986	−174.98 (−332.14, −17.82)	2.18 (0.03)	0% (0.46)
Length of Stay (LOS) (Barnes et al. 2012; Cohen et al. 2002; Coleman et al. 2006; Counsell et al. 2000; Covinsky et al. 1997; Phibbs et al. 2006; Saltvedt et al. 2006, 2002, 2004, 2005; Tibaldi et al. 2009; Wald et al. 2011)	6242	−0.31 (−1.01, 0.38)	0.88 (0.38)	90% (< 0.001)
Outliers Removed	3490	−1.11 (−1.39, −0.83)	7.70 (< 0.001)	15% (0.32)

^a^Risk ratios reported for all meta-analyses except cost and LoS, for which weighted mean differences (WMDs) were reported. ^b^Costs conversions were performed July 10, 2021, using a web-based cost converter endorsed by the Cochrane Economics Methods Group (Cumpston et al. 2019). *N* = Total Number of Patients, CI = Confidence Interval

### Functional outcomes and activity of daily living

The functional decline at discharge was reported in five studies (Barnes et al. 2012; Counsell et al. 2000; Landefeld et al. 1995; McCusker et al. 2001; Westgard et al. 2020). In which, included interventions in the reported studies were the ACE model (Barnes et al. 2012; Counsell et al. 2000; Landefeld et al. 1995), a standardized patient-centered geriatric nursing assessment with a core interdisciplinary team (McCusker et al. 2001), and a comprehensive geriatric assessment (CGA)-based model of care as part of a geriatric acute medical ward with a core interdisciplinary team (Westgard et al. 2020). Meta-analysis of these five studies showed patients who received treatments from the interventions were less likely (but not significant) to experience changes in functional decline at discharge than those who received conventional care (RR = 0.94, 95% CI = 0.87–0.01; *P* = 0.11). ADL was reported in ten studies (Asplund et al. 2000; Barnes et al. 2012; Counsell et al. 2000; Landefeld et al. 1995; Reuben et al. 1995; Saltvedt et al. 2006, 2002, 2004, 2005; Westgard et al. 2020). Individuals receiving geriatric-specific care experienced meaningful but not significant improvement of ADL (RR = 1.09, 95% CI = 0.97–1.22; *P* = 0.01), even after removing two studies (Asplund et al. 2000; Landefeld et al. 1995), to resolve the statistical heterogeneity (Table 4). Additionally, while the included interventions in the reported studies for ADL were similar to that of functional decline, a geriatric evaluation medical (GEM) model of care as part of the hospital wards with a core interdisciplinary team was also included when analyzing ADL.

### Mortality and case fatality

Mortality was reported in 14 studies (Asplund et al. 2000; Barnes et al. 2012; Buurman et al. 2016; Cohen et al. 2002; Counsell et al. 2000; Covinsky et al. 1997; Kircher et al. 2007; Landefeld et al. 1995; Legrain et al. 2011; Saltvedt et al. 2006, 2002, 2004, 2005; Tibaldi et al. 2009). Although these studies indicated that individuals receiving geriatric-specific interventions were less likely to be at risk of mortality, no significant and meaningful differences were found in mortality at discharge between interventions and conventional care (RR = 0.92, 95% CI = 0.79–1.07, *P* = 0.29). Mortality was associated with the following interventions including an ACE model (Barnes et al. 2012; Counsell et al. 2000; Covinsky et al. 1997; Landefeld et al. 1995), an acute geriatrics-based unit (AGU) model with a core interdisciplinary team (Asplund et al. 2000; Legrain et al. 2011), a CGA-based care model as a transitional care bridge program (Buurman et al. 2016), a GEM model with a core interdisciplinary team (Cohen et al. 2002; Kircher et al. 2007; Saltvedt et al. 2006, 2002, 2004, 2005; Tibaldi et al. 2009). Case fatality at discharge was reported in six studies (Asplund et al. 2000; Buurman et al. 2016; Counsell et al. 2000; Covinsky et al. 1997; Landefeld et al. 1995; Tibaldi et al. 2009). Meta-analysis of these six studies indicates that individuals receiving geriatric-specific interventions are less likely to be at risk of fatality at discharge (RR = 0.87, 95% CI = 0.72–1.05, *P* = 0.14), but it is not significant. Case fatality at 1-, 3-, 6-, and 12-month follow-ups were reported in nine studies (Asplund et al. 2000; Counsell et al. 2000; Covinsky et al. 1997; Kircher et al. 2007; Landefeld et al. 1995; Saltvedt et al. 2006, 2002, 2004, 2005). No significant differences were shown between interventions and conventional care in case fatality at follow-up (RR = 0.97, 95% CI = 0.87–1.08, *P* = 0.60). Interventions associated with case-fatality were as follows; AGU model (Asplund et al. 2000), ACE model (Counsell et al. 2000; Covinsky et al. 1997; Landefeld et al. 1995), and GEM model (Kircher et al. 2007; Saltvedt et al. , 2002, 2004, 2005, 2006).

### Discharge destination

Eight studies reported whether patients were discharged home or to a nursing home (Asplund et al. 2000; Barnes et al. 2012; Buurman et al. 2016; Counsell et al. 2000; Covinsky et al. 1997; Landefeld et al. 1995; Tibaldi et al. 2009; Wald et al. 2011). A significant and meaningful effect was identified from the meta-analysis of the eight studies, which was in favor of usual care (RR = 1.06, 95% CI = 1.01–1.12, *P* = 0.02). Seven studies reported whether patients were discharged to their home or a nursing home with a 3-, 6-, and 12-month follow-ups (Asplund et al. 2000; Counsell et al. 2000; Covinsky et al. 1997; Kircher et al. 2007; Landefeld et al. 1995; Saltvedt et al. 2006, 2002, 2004, 2005). Although the meta-analysis of the 12-month follow-up sub-group showed a nonsignificant result (RR = 0.93, 95% CI = 0.81–1.07; *P* = 0.30), the overall effect was significant and meaningful but in favor of usual care (RR = 1.11, 95% CI = 1.03–1.20; *P* = 0.008). The following interventions are associated with discharge destination: the ACE model (Barnes et al. 2012; Counsell et al. 2000; Covinsky et al. 1997; Landefeld et al. 1995; Wald et al. 2011), the CGA model (Buurman et al. 2016), and GEM model (Kircher et al. 2007; Saltvedt et al. , 2002, 2004, 2005; 2006, Tibaldi et al. 2009).

### Hospital readmissions

Hospital readmissions were reported in eleven studies (Asplund et al. 2000; Barnes et al. 2012; Buurman et al. 2016; Coleman et al. 2006; Counsell et al. 2000; Kircher et al. 2007; Landefeld et al. 1995; Legrain et al. 2011; Reuben et al. 1995; Tibaldi et al. 2009; Wald et al. 2011). Meta-analysis of these eleven studies identified no significant difference in hospital readmissions within 1-, 3-, and 6-month of discharge between individuals receiving the geriatric-specific intervention and conventional care (RR = 0.98, 95% CI = 0.88–1.09; *P* = 0.75). Significant statistical heterogeneity was observed and resolved by removing two studies (Buurman et al. 2016; Reuben et al. 1995), as outliers, but the effect remained nonsignificant (RR = 0.98, 95% CI = 0.87–1.11; *P* = 0.78). The AGU model (Asplund et al. 2000; Legrain et al. 2011), the ACE model (Barnes et al. 2012; Counsell et al. 2000; Landefeld et al. 1995; Wald et al. 2011), the CGA model (Buurman et al. 2016), a hybrid care transition intervention (Coleman et al. 2006), GEM model (Kircher et al. 2007; Tibaldi et al. 2009), and a patient assessment using a standardized, multidimensional assessment instrument (Reuben et al. 1995).

### Length of hospital stay

LoS was reported in 12 studies (Barnes et al. 2012; Cohen et al. 2002; Coleman et al. 2006; Counsell et al. 2000; Covinsky et al. 1997; Phibbs et al. 2006; Saltvedt et al. 2006, 2002, 2004, 2005; Tibaldi et al. 2009; Wald et al. 2011). Meta-analysis of these studies showed that geriatric patients receiving geriatric-specific interventions experienced shorter but nonsignificant LoS than those receiving conventional care (WMD =  − 0.31, 95% CI =  − 1.01–0.38; *P* = 0.38). Significant heterogeneity was observed between studies for this comparison. After removing seven studies (Coleman et al. 2006; Counsell et al. 2000; Saltvedt et al. 2006, 2002, 2004, 2005; Wald et al. 2011), the meta-analysis showed that patients receiving interventions experienced significantly shorter LoS (WMD =  − 1.11, 95% CI =  − 1.39 to − 0.83; *P* < 0. 001), with statistical heterogeneity resolved. Included interventions associated with LoS in this study are the ACE model (Barnes et al. 2012; Counsell et al. 2000; Covinsky et al. 1997; Wald et al. 2011); GEM model (Cohen et al. 2002; Phibbs et al. 2006; Saltvedt et al. 2002, 2004, 2005, 2006; Tibaldi et al. 2009), a hybrid care transition intervention (Coleman et al. 2006).

### Costs

Nine studies reported hospital costs (Asplund et al. 2000; Barnes et al. 2012; Cohen et al. 2002; Coleman et al. 2006; Counsell et al. 2000; Covinsky et al. 1997; Phibbs et al. 2006; Tibaldi et al. 2009; Wald et al. 2011). The meta-analysis of the studies indicated that the costs of geriatric-specific models were nonsignificantly less than the costs of conventional care (WMD =  − $401.13, 95% CI =  − $821.43 to + $19.17; *P* = 0.06). Significant statistical heterogeneity was observed and resolved by removing one outlier (Cohen et al. 2002). The results of the meta-analysis confirmed that the costs of geriatric-specific interventions were significantly less than those of conventional care (WMD =  − $174.98, 95% CI =  − $332.14 to − $17.82; *P* = 0.03). Interventions associated with costs were; AGU model (Asplund et al. 2000), ACE model (Barnes et al. 2012; Counsell et al. 2000; Covinsky et al. 1997; Wald et al. 2011), GEM model (Cohen et al. 2002; Phibbs et al. 2006; Tibaldi et al. 2009), a hybrid care transition intervention (Coleman et al. 2006).

## Discussion

This is the first study to analyze geriatric-specific interventions based on all or part of the intervention components for around a decade and is the first review including a meta-analysis of RCTs in an acute hospital-wide setting (Bakker et al. 2011; Baztan et al. 2009; Fox et al. 2012). Results from meta-analyses demonstrate that acute geriatric pathways introduced during the acute illness phase have significantly beneficial effects over conventional care in reducing costs, and LoS. No differences were found in functional decline, ADL, mortality, case fatalities at discharge, case fatality at follow-up, discharge destination at discharge and follow-up, or hospital readmissions. Similar to an earlier review assessing the effects of hospital-wide interventions for older inpatients, a single best practice could not be described to improve the quality of care and effectiveness (Bakker et al. 2011). The heterogeneity in the studies can be explained by the nature of the care package and intervention components. All the eligible studies had at least one of the mentioned geriatric-specific models of care components. Although interventions may be applied at different points of patient's journey, one of the primary strengths of this study is the broad comparison of the interventions representing the whole patient journey based on the wide variety of the inclusion criteria considering the components of the intervention care models. In contrast, due to summarizing the studies with a broad perspective, comparability limitations of the interventions still exist.

Another important finding from this review was the tendency toward positive results which can be identified in a frail older population. From an economic perspective, studies showed that even with small clinical effects, hospitalization costs are lower in geriatric-specific interventions (Asplund et al. 2000; Barnes et al. 2012; Coleman et al. 2006; Covinsky et al. 1997; Tibaldi et al. 2009). Boyd et al. (2009) suggested that the average time for partial or full ADL recovery after hospitalization is 18 months. Hence, studies show a tendency toward less functional decline (Barnes et al. 2012; Counsell et al. 2000; Landefeld et al. 1995; Westgard et al. 2020), less mortality (Buurman et al. 2016; Cohen et al. 2002; Counsell et al. 2000; Saltvedt et al. 2006, 2002, 2004, 2005; Tibaldi et al. 2009), less case fatality (Buurman et al. 2016; Counsell et al. 2000; Covinsky et al. 1997; Landefeld et al. 1995), or readmissions (Barnes et al. 2012; Coleman et al. 2006; Landefeld et al. 1995; Legrain et al. 2011; Tibaldi et al. 2009) are valuable due to showing positive results and health outcomes identified in the frail geriatric population.

The RCTs in this review selected patients based on their age ($\ge 65$ years). Studies suggest that the frailest older people are the patients who benefit most from geriatric-specific pathways, regardless of the condition(s) leading to admission (Baztan et al. 2009; Ellis et al. 2017; Malone et al. 2014). Although the analyses did not provide this conclusion, given the characteristics of patients, it can be concluded that these findings are mainly applicable to septuagenarians and octogenarians admitted through emergency departments with acute illnesses or other complex morbidities (Fox et al. 2012). The findings of this study have relevance for researchers, clinicians, and policymakers. The findings show that partial or full implementation of these models can have moderate to significant effects on acute older persons' health outcomes due to patient-centered care, frequent medical review, early rehabilitation, and early discharge planning components (Fox 2013). Although further research is required, since interdisciplinary teams are considered an essential component of geriatric-specific models, it is suggested that clinicians consider a multidisciplinary team of geriatricians, geriatric nurses, physiotherapists, occupational therapists, dieticians, and social workers to improve the care of older patients (Hickman et al. 2007).

Hospital administrators should consider the success of older person-specific interventions for acute hospital-wide settings in decreasing Length of Stay (LoS) followed by a decrease in cost due to saving bed days and utilization of a multidisciplinary team. They should also pay attention to improving patient flow and decreasing long-term care placement (which results in saving more cost). Accordingly, cost-saving and shorter stays can be achieved by investing in hospital-wide geriatric-specific models to deal with the increasing number of complex older patients admitted to hospitals. As per findings of the meta-analysis patients receiving interventions experienced significantly shorter LoS, i.e., at least 1 day, and less costly than those of conventional care, around approximately $175. Although the initial cost for adding a functional geriatric-specific pathway is low, with the expected increase in age demographics and the impact of older patients' health problems as measured by financial cost, mortality, morbidity, and resources, the need for hospital-wide geriatric-specific pathways becomes vital. Cost-effectiveness and efficiency of such hospital-wide geriatric-specific interventions require further research. Policymakers should, therefore, pay closer attention to the associations between a tendency to lower costs and positive health outcomes by successfully implementing a geriatric-specific pathway as a business-as-usual setting. Considering the fact that examination of patient-relevant outcomes such as well-being or quality of life has become increasingly important in older age, successful implementation of these interventions results in smoother patient flow through the utilization of efficient admission and discharge processes, building an effective professional staffing model, and providing ongoing education on geriatric-specific principles (Fox et al. 2012).

### Comparison with previous systematic reviews and meta-analyses

While previous studies have analyzed geriatric-specific interventions most often based on all or part of the intervention components (Bakker et al. 2011; Baztan et al. 2009; Fox et al. 2012), to the best of the authors' knowledge, this is the first study analyzing geriatric-specific interventions based on a meta-analysis of RCTs in an acute hospital-wide setting. The most recent systematic review and meta-analysis of geriatric-specific interventions was conducted around a decade ago by Fox et al. (2012, 2013). Furthermore, only one other systematic literature review was conducted by Bakker et al. (2011) in an acute hospital-wide setting, in which a meta-analysis was not performed and both RCT and controlled clinical trials were considered eligible in the systematic review. Furthermore, while the inclusion criteria of the current study were similar to that of Bakker et al. (2011), Baztan et al. (2009), Fox et al. (2012), this study can be considered as a rigorous and important update considering only RCT studies in an acute hospital-wide setting after a decade to the previous reviews adding. Baztan et al. (2009) analyzed 11 studies where functional decline at discharge and living destination at discharge (at home) were significant and in favor of intervention, and with no differences in case fatality, readmission, LoS, and costs. Furthermore, Fox et al. (2012) performed a systematic review and meta-analysis of 13 studies, in which acute geriatric unit care was associated with fewer falls, less delirium, less functional decline at discharge, shorter LoS, fewer discharges to a nursing home, lower costs, and more discharges to home. Accordingly, no significant differences were found in functional decline between baseline hospital admission status and discharge, mortality, or hospital readmissions. While the result of the current meta-analysis was similar to that of the study of Fox et al. (2012), we did not analyze falls, pressure ulcers. Furthermore, an unexpected and contradictory result from this study regarded to discharge destination which was in favor of usual care, whereas in the previous studies, it was in favor of the intervention (Baztan et al. 2009; Fox et al. 2012), or had not significant effect (Bakker et al. 2011). Ultimately, while the result of this study regarding costs and LoS was similar to that of Fox et al. (2012) findings, Baztan et al. (2009) and Bakker et al. (2011) found small or no effects on these measures.

## Limitations

This review highlights the limited number of studies using RCTs to investigate the effectiveness of geriatric-specific models. Missing data were minimized since study authors were contacted to provide unpublished data. However, one of the limitations of this study is that the publication bias was not minimized because non-RCT and unpublished studies were not analyzed. Therefore, it is recommended to also consider non-RCT, whitepapers and study registries for the future development of this research study. Furthermore, since the number of RCTs included is small, and most of the trials were between 2000 and 2009, the ability to draw conclusions is restricted regarding estimations of model effectiveness in modern-day hospitals. A recent review argued that the lack of RCTs since 2000 is "…due to acute geriatric units having become the norm for care, so that there are presently no standard hospital care units with which they can be compared…" (Baztan et al. 2009) (Page 8). According to other reviews (Bakker et al. 2011; Ellis et al. 2017; Fox et al. 2012); including RCTs (Buurman et al. 2016; Westgard et al. 2020) and non-RCTs (Fox et al. 2013), this may not be the case, because acute geriatric-specific interventions are far from being prevalent in hospital-wide settings, and recent studies have repeatedly recommended reviewing the effectiveness of this type of care (Boockvar et al. 2020; Lund et al. 2021; Robert et al. 2020). Furthermore, a contradictory and unexpected finding extracted from the meta-analysis of this study was regarding to discharge destination which was in favor of usual care. This unexpected finding is directly related to the selection of the number of studies, in this case only RCTs. Since the number of RCTs included is small, and previous studies showed significant and meaningful findings regarding discharge destination (Bakker et al. 2011; Ellis et al. 2017; Fox et al. 2012), the ability to draw conclusions is restricted and more research is required. As older people and their health trajectories are very heterogeneous, the sample structures and information on severity of the diagnosis can be compared between the included studies and those with differing results.

Most of the earlier trials were restricted to admission through the emergency department (Baztan et al. 2009; Fox et al. 2012). Given the importance of hospital-wide interventions (Bakker et al. 2011), future studies should include other forms of admission and, indeed the impact of older person-specific units for surgical patients. Similar to earlier meta-analyses (Baztan et al. 2009; Fox et al. 2012), this study found a significant effect on reducing costs. In contrast to an earlier meta-analysis (Baztan et al. 2009), which found nonsignificant effects on LoS, the result of this study showed significant reductions in LoS. While this systematic review included a diverse group of participants in a hospital-wide setting, heterogeneity was low in most meta-analyses, supporting the validity of the results. Since not all studies analyzed in this review provided data on the components of the interventions, a firm conclusion could not be drawn on the effect of interventions in the hospital-wide setting. Future studies should review a larger sample size to evaluate the effectiveness of older person-specific interventions and their components including medical review, early rehabilitation, early discharge planning, prepared environment, and patient-centered care. Furthermore, one of the limitations of the current study is that although this systematic review included a diverse group of participants, a sub-group analysis was not performed. The primary reason sub-group analysis was not performed is; "…Although issues related to subgroup analysis have been debated for decades and numerous guidance on subgroup analyses has been advocated, controversy remains regarding the conduct, reporting, and interpretation of subgroup analyses in clinical trials…studies found that there has been no improvement in the reporting of sub-group analyses, and that there were discrepancies between subgroup analyses planned in protocols and journal publications of clinical trials…" (Fan et al. 2019) (Page 2).

## Conclusions

The population of older people is increasing drastically in almost every country in the world, and the developing world is going grey. With this trend in the population, developments in hospital care explicitly aimed to improve care for all frail older patients, and changes in policies across health settings will improve the likelihood of geriatric care model optimization and implementation throughout the hospitals. Although evidence from the literature showed the availability of various older person-specific models, only a few hospital-wide RCT studies were identified. However, due to a crucial need for hospital-wide interventions, it is important to establish scientific standards in this setting supporting patients. As the proportion of acute geriatric patients progressively increases, such evidence-based investigation of interventions should be a key focus for health research. Therefore, further research is required in order to study hospital-wide geriatric-specific models and alternative approaches for improvements in hospital-wide care for frail older patients.

## Supplementary Information

Below is the link to the electronic supplementary material.

Supplementary file1 (DOCX 319 KB)

## Footnotes

1 While in this study the concept of patient-centered care was defined based on that of Fox et al. (2012) in their respective review, when consulting the literature on efforts to conceptualize patient-centered care and what is a current understanding of PCC, there might also be other explanations. For instance, Scholl et al. (2014) defined patient-centered care by elements such as shared decision-making, emotional support and patient empowerment, or inclusion of the patient's environment. In other hand, patient-centered care is typically defined as the ideal goal and concept of providing care.
