# Supplementary material for: Efficacy of acute care pathways for older patients: a systematic review and meta-analysis
Source: Eur J Ageing. 2022 Nov 17;19(4):1571–85. doi: 10.1007/s10433-022-00743-w (PMC9729482; doi:10.1007/s10433-022-00743-w)
Supplement: Supplementary file 1 — Supplementary file1 (DOCX 319 KB) [file 10433_2022_743_MOESM1_ESM.docx]

**Appendix (S1):** Search strategy and subject headings.

A subject librarian provided advice for the literature search with input from team members to pinpoint medical subject headings reflective of the inclusion criteria which could define the effectiveness and efficiency of the geriatric-specific models. Electronic databases searched included EMBASE, PubMed, CINAHL, OECD health policies and data, NHSEED, Web of Science, SCOPUS, SSRN, Cochrane Library, ProQuest, ScienceDirect, SpringerLink and Sage, and Wiley. Further searches were undertaken using text word search within the abstracts and titles of the mentioned databases and in high-impact journals in the field of gerontology, including the Journal of the American Geriatrics Society (JAGS), Archives of Gerontology and Geriatrics, Age and Ageing, Journal of the American Medical Association, Gerontologist, Journals of Gerontology, and bibliography information of comprised studies and previous reviews aligned with the inclusion criteria. The last update for the current review was performed on October 5, 2021. Full-text papers of eligible papers were retrieved for further review, and in cases of insufficient information in the title and abstract, the complete article was reviewed. It should be noted that for the eligible and included research studies forward citation search was also included.

**Limits based on the eligibility criteria:**

Published Date: 1995 - 2021

Age Groups: Age $\geq65$

Language: English

Study Types: Eligible studies for the current review included randomised controlled trial (RCT) studies that compared acute geriatric-specific models with usual care in a hospital-wide setting.

**Subject Heading & MeSH Searches (For Ovid Databases (e.g., MedLine, EMBASE), CINHAL)^[[1]](#footnote-1)^:**

**Study Population:** Elderly — Aged — 65 Years and Over — Aged,65 Years and Over — Older Patient(s) — Frail Elderly — Geriatrics — Geriatric Patient(s)

**Geriatric Models of Care, Assessment, Aftercare, and Programs:** Comprehensive Health Care — exp Geriatric Assessment — Critical Care — exp Acute Care for Elderly — exp Health Services for the Aged — Aftercare — Acute Medical Care — Patient Care Planning — Healthcare Planning — exp Acute Geriatric Unit — Advance Care Planning — Critical Pathways — Patient Pathway — Aftercare Program — Critical Care Outcomes — Managed Care Programs — Health Status

**Hospital Units, Wards and Centres:** Hospitals — Acute Disease — Psychiatric Department, Hospital — Hospitals, Psychiatric — Emergency Medicine — Internal Medicine — Rehabilitation Centres — exp Geriatric Assessment — exp Geriatricians — Intensive Care Units — Coronary Care Units — Respiratory Care Units — Hospital Units — Emergency Medical Services — Emergency Service, Hospital — Trauma Centres — Dental Care for Aged — Housing for the Elderly — exp Health Services, Geriatric — Health Services for the Elderly — Geriatric Health Services

**Acute Conditions and Complications:** Hospital Mortality — Mortality — exp Infections — exp Bacterial Infections — exp Mycoses — exp Virus Diseases — exp Parasitic Diseases — exp Acute Disease — Asymptomatic Diseases — Asymptomatic Infections — Catastrophic Illness — Chronic Disease — Multiple Chronic Conditions — Communicable Diseases — Blood-Borne Infections — Communicable Diseases, Emerging — Communicable Diseases, Imported — Sexually Transmitted Diseases — Convalescence — Critical Illness — Disease Progression — Clinical Deterioration — Remission, Spontaneous — Disease Resistance — Disease Susceptibility —Genetic Predisposition to Disease — Emergencies — Facies — Iatrogenic Disease — Cross Infection — Late Onset Disorders — Neglected Diseases — Noncommunicable Diseases — Rare Diseases — Recurrence — Reinfection — Symptom Flare Up — Undiagnosed Diseases — Acantholysis — exp Arrhythmias, Cardiac — exp Death — Dehydration — Acute Disease — Frailty — Long Term Adverse Effects — exp Postoperative Complications — Parasitic Diseases — exp Nervous System Diseases — exp Eye Diseases — exp Urogenital Diseases — exp Cardiovascular Diseases — exp Congenital Abnormalities — exp Fetal Diseases — exp Genetic Diseases, Inborn — exp Skin Diseases — exp Skin and Connective Tissue Diseases — exp Metabolic Diseases — exp Nutrition Disorders — exp Immune System Diseases — exp Muscular Atrophy — Sarcopenia — exp Atrophy — Ethnopsychology — exp Pain — Accidental Falls — Immobilization — exp Mental Disorders — exp Cognition Disorders — exp Cognitive Dysfunction — Delirium — Confusion — exp Neurocognitive Disorders — Depression — Polypharmacy — Drug Therapy — Skin Ulcer — Pressure Ulcer — Malnutrition — Urinary Incontinence — exp Urination Disorders — exp Iatrogenic Disease — Cross Infection — exp Occupational Diseases — exp Sleep Disorder — exp Sleep Disorders, Intrinsic — Dizziness — Mobility Limitation — exp Hearing Loss — exp Vision Disorders — exp Nervous System Diseases

**Cost, Cost Analysis, Efficiency, Effectiveness, Healthcare Delivery and Policy:** Health Care Economics and Organizations — exp Health Planning — Health Care Rationing — Health Care Reform — Health Plan Implementation — Health Planning Guidelines — Health Planning Technical Assistance — Health Priorities — Health Resources — Health Services Research — National Health Programs — Regional Health Planning — Home Care Agencies — Policy — exp Patient Care Management — exp Delivery of Health Care — exp Health Care Quality, Access, and Evaluation — exp Health Care Quality, Access, and Evaluation — exp Health Services Research — Health Services Needs and Demand — Health Services for the Aged — Geriatric Health Services — Health Services for the Elderly — Health Services, Geriatric — Geriatric Health Service — Health Service, Geriatric — Service, Geriatric Health — Services, Geriatric Health — exp Costs and Cost Analysis — Cost-Benefit Analysis — Health Care Costs — Direct Service Costs — Drug Costs — Employer Health Costs — Hospital Costs — exp Delivery of Health Care, Integrated — exp Health Care Evaluation Mechanisms — exp Quality of Health Care — exp Health Services Administration — Universal Health Care — Administration, Health Services — Efficiency — exp Organization and Administration — exp Costs and Cost Analysis

**Rehabilitation:** exp Therapeutics — Activities of Daily Living — Psychiatric Rehabilitation — Functional Status — Cardiac Rehabilitation — Correction of Hearing Impairment — Communication Methods, Total — Manual Communication — Early Ambulation — Neurological Rehabilitation — Stroke Rehabilitation — Occupational Therapy — Physical Therapy Modalities — Rehabilitation, Vocational — Telerehabilitation — Recovery of Function — Self Care — Nutrition Therapy — Accident Prevention — Primary Prevention — Secondary Prevention — Safety Management — exp Patient Care — exp Critical Care — Long-Term Care — Intensive Care — Cardiovascular Nursing — Critical Care Nursing — Developmental Disability Nursing — Emergency Nursing — Geriatric Nursing —Holistic Nursing — Home Nursing — Respite Care — Hospice and Palliative Care Nursing — Neonatal Nursing — Medical-Surgical Nursing — Nephrology Nursing — Neuroscience Nursing — Nursing, Practical — Obstetric Nursing — Occupational Health Nursing — Oncology Nursing — Primary Nursing — Psychiatric Nursing — Rehabilitation Nursing — Trauma Nursing — Patient Positioning — Occupational Therapy — exp Rehabilitation — Physical Medicine — Nursing Assessment — Nursing, Team — Nursing Process — Patient Care Planning — Case Management — Critical Pathways — Social Work — Pharmacists — exp Quality of Health Care — exp Administration, Health Services — Risk Management — Point-of-Care Systems — Utilization Review

**Hospitalization, Discharge, and Patient Care:** Patient Care — Ambulatory Care — Continuity of Patient Care — Aftercare — Patient Discharge — Patient Transfer — Retention in Care — Transitional Care — Delayed Diagnosis — Duration of Therapy — Episode of Care — Length of Stay — Hospitalization — Patient Admission — Patient Readmission — Institutionalization — exp Life Support Care — Long-Term Care — Nursing Care — Home Nursing — Primary Care Nursing — Palliative Care — Subacute Care — Terminal Care — Hospice Care — Continuity of Patient Care — exp Comprehensive Health Care

*** Keywords were extracted based on the subject headings (combination of the subject headings).**

| **Table S1.** Descriptive characteristics of studies included in the systematic literature review and meta-analysis. | | | | | | |
| --- | --- | --- | --- | --- | --- | --- |
| **Study Author Info (Year) [Ref], Country** | **Design, Setting, Time of Assessment and Participants** | **Comparison Groups** | | **Comparison Units** | | **Primary/Secondary Outcomes** ¶ **and Results (Effect, P-Value)** § |
|  |  | **Intervention (Acute Geriatric Unit/Model)** | **Control (Usual/Conventional Care)** | **Intervention** | **Control** |  |
| Landefeld, C. S., et al. (1995) [1], USA * | RCT, assessment at hospital admission, follow-up 3 months, and discharge. All patients $\geq$ 70 years, admitted to emergency department, not admitted (not requiring upon admission) to a speciality unit including intensive care, cardiology, oncology, telemetry at a university hospital. All patients were acutely admitted for general medical care and support. | n=327, mean age=80.2$\pm$6.9, male=32%, female=68%, 7% living in a long-term care institution, 33% living alone in a private home, 27% have poor overall health status two weeks before admission, 36% have poor overall health status on admission, IADL on admission = 4.2$\pm$2.4. Major diagnostic symptoms: 11% neurological, 18% cardiac, 18% infection, 19% pulmonary, 19% gastrointestinal, 14% others. Comorbidities: 26% CHF, 23% cancer, 22% CLD, 17% history of myocardial infarction, 12% cerebrovascular disease, 10% dementia. | n=324, mean age=80.1$\pm$6.6, male=35%, female=65%, 9% living in a long-term care institution, 34% living alone in a private home, 28% have poor overall health status two weeks before admission, 44% have poor overall health status on admission, IADL on admission = 4.1$\pm$2.5. Major diagnostic symptoms: 13% neurological, 15% cardiac, 20% infection, 14% pulmonary, 20% gastrointestinal, 18% others. Comorbidities: 23% CHF, 21% cancer, 20% CLD, 21% history of myocardial infarction, 18% cerebrovascular disease, 13% dementia. | ACE unit specifically designed for the study with a core team of geriatricians, registered nurses, social workers, physiotherapists, and nutritionists. Frequent medical review to minimize the adverse effects of procedures and medications; early discharge planning with the goal of returning patients to their home; prepared environment e.g., uncluttered hallways, large clocks and calendars, and handrails; patient-centred care and early rehabilitation with an emphasis on independence and early rehabilitation. | Usual care is in another medical ward. Attending physicians, resident physicians and nurses provided care to patients in both the intervention and usual-care groups. Hence, similar general support and services provided to the conventional care and usual care group. | Functional outcomes (+, p=0.03), Institutionalisation (+, no p-val), Psychological and health outcomes (+, p > 0.02), ADL (+, p=0.06), LOS (+, p=0.4), Discharge Destination (+, no p-val), Functional Outcomes (time of discharge) (+, p-val 0.06), Costs (+, p=0.3), Case Fatality (+, p=0.1) |
| Reuben, D. B., et al. (1995) [2], USA | RCT, assessment at hospital admission, follow-up 3 and 12 months, and discharge. All patients $\geq$ 65 years in whom at least 1 of the following criteria were present: stroke, immobility, impairment in any basic ADL, malnutrition, incontinence, confusion or dementia, prolonged bed rest, recent falls, depression, social or family problems, an unplanned readmission to the hospital, a new fracture, and age $\geq$80 years. Patients were excluded if admitted for terminal care, were not members of the health plan, were discharged/died before randomization, did not speak English, or were admitted from a nursing home. | n=1337, mean age=77.6, male=44%, female=56%, 2.24% live alone, baseline basic ADL score = 85.1, 3 months basic ADL score = 80.5, 12 months basic ADL score = 83.4, 74% survival rate at 12 months, 31% unplanned readmission within 3 months, 21.05% with confusion or dementia, 19.75% prolonged bed rest, 14.77 immobility and falls within 3 months. | n=1016, mean age=76.7, male=52%, female=48%, 2.85% live alone, baseline basic ADL score = 86.0, 3 months basic ADL score = 80.2, 12 months basic ADL score = 83.7, 75% survival rate at 12 months, 36.5% unplanned readmission within 3 months, 23.12% with confusion or dementia, 19.44% prolonged bed rest, 13.53 immobility and falls within 3 months. | Patient assessment using a standardized, multidimensional assessment instrument used by nurse practitioner along with a limited physical examination focusing on geriatric issues, then social workers assessed functional status and cognitive and emotional health; early involvement of the geriatrician to discuss the case with the entire team. Medical care review and patient-centred care by geriatrician and primary care physician. Team conferences were held daily. | Patients assigned to usual care or the control group were eligible to receive all standard and usual treatments and services ordered and provided by their primary physicians and nurses. | ADL (NS, no p-val), Social Activities (NS, no p-val), Mental Health (+, p-val = 0.04), Mortality (NS, no-p-val), LOS (NS, no pval), Readmission (+, no p-val) |
| Covinsky, K. E., et al. (1997) [3], USA * | RCT, assessment at hospital admission, follow-up 3 months, and discharge. All patients $\geq$ 70 years, admitted to emergency department, not admitted (not requiring upon admission) to a speciality unit including intensive care, cardiology, oncology, telemetry) at a university hospital. All patients were acutely admitted for general medical care and support. | n=326, mean age=80, male=32%, female=68%, mean comorbidity score = 2, 38% independent in ADL, total cost including intervention = $6608$\pm$$6551, 36.7% 90-day readmission, 24.1% 90-day nursing home use. Diagnostic symptoms: 11% neurological, 18% cardiac, 18% infection, 19% pulmonary, 19% gastrointestinal, 14% others. Comorbidities: 26% CHF, 23% cancer, 22% CLD, 17% history of myocardial infarction, 12% cerebrovascular disease, 10% dementia. | n=324, mean age=80, male=35%, female=65%, mean comorbidity score = 2, 41% independent in ADL, total cost for usual care = $7240$\pm$$8504, 41.1% 90-day readmission, 32.3% 90-day nursing home use. Major diagnostic symptoms: 13% neurological, 15% cardiac, 20% infection, 14% pulmonary, 20% gastrointestinal, 18% others. Comorbidities: 23% CHF, 21% cancer, 20% CLD, 21% history of myocardial infarction, 18% cerebrovascular disease, 13% dementia. | ACE unit specifically designed for the study with a core team of geriatricians, registered nurses, social workers, physiotherapists, and nutritionists. Frequent medical review to minimize the adverse effects of procedures and medications; early discharge planning with the goal of returning patients to their home; prepared environment e.g., uncluttered hallways, large clocks and calendars, and handrails; patient-centred care and early rehabilitation with an emphasis on independence and early rehabilitation. | Usual care is in another medical ward. Attending physicians, resident physicians and nurses provided care to patients in both the intervention and usual-care groups. Hence, similar general support and services provided to the conventional care and usual care group. | Functional outcomes (+, p=0.03), Institutionalisation (+, no p-val), ADL (+, p=0.06), LOS (+, p=0.4), Discharge Destination (+, no p-val), Functional Outcomes (time of discharge) (+, p-val 0.06), Case Fatality (+, p=0.1), total direct cost (+, p=0.742), indirect cost (+ p=0.625), total cost (+, p=0.926), 90-day readmission (+, p=0.283) |
| Counsell, S. R., et al. (2000) [4], USA | RCT, assessment at hospital admission, follow-up 1, 3, 6 and 12 months, and discharge. All patients (community-dwelling persons) $\geq$ 70 years, admitted to a medicine or family practice service, at a community teaching hospital. Patients were excluded if admitted to intensive care, cardiology, oncology, telemetry, not residing in a nursing home, and LOS $<$ 2 days or previously enrolled in study. | n=767, mean age=80$\pm$7, male=40%, female=60%, 33% living alone, 30% living with another adult, 29% very good and excellent overall health status upon admission, 19% poor overall health status upon admission, 31% improved ADL (admission to discharge). Major diagnostic symptoms: 19% neurological, 11% cardiac, 14% infection, 25% pulmonary, 20% gastrointestinal, 12% others. Comorbidities: 30% CHF, 9% cancer, 27% CLD, 17% history of myocardial infarction, 21% cerebrovascular disease, 16% dementia. | n=764, mean age=79$\pm$7, male=39%, female=61%, 31% living alone, 28% living with another adult, 29% very good and excellent overall health status upon admission, 18% poor overall health status upon admission, 31% improved ADL (admission to discharge). Major diagnostic symptoms: 21% neurological, 12% cardiac, 14% infection, 23% pulmonary, 17% gastrointestinal, 12% others. Comorbidities: 28% CHF, 7% cancer, 21% CLD, 17% history of myocardial infarction, 22% cerebrovascular disease, 18% dementia. | ACE unit designed for the study with a core team of geriatrician, registered nurse, social worker, and physiotherapist. Designed with frequent medical review to minimize the adverse effects of procedures and medications; early discharge planning with the goal of returning patients to their home; prepared environment e.g., uncluttered hallways, large clocks and calendars, and handrails; patient-centred care and early rehabilitation with an emphasis on independence and early rehabilitation. | Patients assigned to usual care or the control group were eligible to receive all standard and usual treatments and services ordered and provided by their primary physicians and nurses. Geriatric consultation team is also already available in the hospital and may be available for usual care. | ADL (+, p=0.027), LOS (NS), Costs (NS), Home healthcare visits (NS), Readmissions (NS), Mortality (NS), Institutionalisation (+, no P-val), Mobility (NS), Functional Outcomes (NS), Case Fatality (+, no P-val) |
| Asplund, K., et al. (2000) [5], Sweden | RCT, assessment at hospital admission, follow-up 3 months, and discharge. All patients $\geq$ 70 years, admitted to emergency department for general medical care at a university hospital. Patients were excluded if admitted to a speciality unit including intensive care, cardiology, oncology, telemetry). All patients were acutely admitted from ED to general medical wards for general medical care and support. | n=190, mean age=80.9, male=42%, female=58%, 46% living alone, 16% living in institution, 47% impaired cognitive function at entry, 52% independent in ADL, 71% discharged to home, 80% returning to previous living. History and major diagnostic symptoms: 24% myocardial infarction, 25% angina pectoris, 13% cardiac failure, 16% stroke, 17% diabetes, 6% dementia, 21% chest pain, 8% other pain, 18% dyspnoea, 11% nausea/vomiting, 11% vertigo. | n=223, mean age=81.0, male=37%, female=63%, 51% living alone, 16% living in institution, 53% impaired cognitive function at entry, 44% independent in ADL, 64% discharged to home, 74% returning to previous living. Major diagnostic symptoms: 17% myocardial infarction, 27% angina pectoris, 15% cardiac failure, 22% stroke, 23% diabetes, 5% dementia, 25% chest pain, 13% other pain, 18% dyspnoea, 11% nausea/vomiting, 11% vertigo. | The acute geriatrics-based ward organised solely for the purpose of the study with a core interdisciplinary team of internist, geriatrician, registered nurse, physiotherapist, occupational therapist, and dietician. Designed with comprehensive assessment, interdisciplinary conferences 1 day/week, early rehabilitation and discharge planning. | Two existing general medical wards. Discharge planning initiated shortly before discharge, and early discharge planning is not provided. Core team of physician, internist, nurse. While available, service from geriatric, dietician, social worker, occupational therapist, and physiotherapist was not provided. | Case fatality (+, no p-val), Institutionalisation (+, no p-val), Functional outcomes (N/ST/U), Psychological wellbeing (+, no p-val), LOS (+, no p-val), Costs (NS, no p-val), Readmission (NS, no p-val) |
| McCusker, J., et al. (2001) [6], Canada | RCT, assessment at hospital admission, follow-up 1 and 4 months, and discharge. All patients $\geq$ 65 years, expected to be released from ED to the community with a ISAR $>$ 2 score and their primary family caregivers, at four university hospitals. Patients were excluded if referred from a nursing home and/or chronic disease hospital, expected by ED staff to require admission, unable to communicate in French or English, not residents of study area, or medically unstable or cognitively impaired and there was no family member to act as proxy. | n=178, mean age=76.7$\pm$7.1, male=44.4%, female=55.6%, 34.8% living alone, 15.2% with severe functional disability. Chief complaint symptom: 14% general, 7.9% mental/nervous, 18.5% cardiorespiratory, 14% digestive, 6.2% genitourinary, 16.9% musculoskeletal, 4.5% others. | n=210, mean age=76.5$\pm$7.0, male=34.8%, female=65.2%, 45.2% living alone, 11.9% with severe functional disability. Chief complaint symptom: 9.5% general, 9.1% mental/nervous, 16.7% cardiorespiratory, 16.2% digestive, 6.2% genitourinary, 16.2% musculoskeletal, 8.6% others. | The primary intervention consists of a brief, standardized patient-centred geriatric nursing assessment with a core interdisciplinary team of experts in geriatric medicine, nursing, and social work. Also, notification of the primary care physician and home care providers, and other referrals will be provided. | Patients assigned to usual care or the control group were eligible to receive all standard and usual treatments and services ordered and provided by their primary physicians and nurses, without disclosure of the screening result. | Functional decline at 4 months and at discharge (+, no p-val), patient and caregiver satisfaction (+ no p-val) |
| Cohen, H. J., et al. (2002) [7], USA ** | MRCT, assessment at hospital admission, follow-up 12 months, and discharge. All patients (frail elderly) $\geq$ 65 years, admitted either to a medical or surgical ward at 11 Veterans affair medical centres, with a frail condition including inability to perform one or more basic ADL, a stroke within the previous three months, a history of falls, difficulty walking, malnutrition, dementia, depression, one or more unplanned admissions in the previous 3 months, prolonged bed-rest, or incontinence. Patients were excluded if they already/previously receiving/received GEM-type care, currently enrolled in trial, a severe disabling/terminal disease, were unable to return for follow-up. | n=346, male=98%, female=2%, cost of initial hospitalisation = $13,499$\pm$621, cost of care after discharge = $22,816$\pm$1,080. | n=348, male=98%, female=2%, cost of initial hospitalisation = $10,758$\pm$592, cost of care after discharge = $26,533$\pm$1,201. | A GEM unit organised for the purpose of the study with a core interdisciplinary team of geriatrician, social worker, and nurse. Designed with specific instructions to complete the history taking and physical examination, including screening for geriatric syndromes, comprehensive assessment; evaluating the patient’s functional, cognitive, affective, and nutritional status; assess the caregiver’s capabilities; and evaluate the patient’s social situation. A patient-centred rehabilitation plan must be developed with preventive and management services. Intervention requires at least one follow-up appointment. | Patients assigned to usual care or the control group were eligible to receive all standard and usual treatments and services ordered and provided by their primary physicians and nurses. | Mortality (NS, no p-val), Total cost (+, p=0.29), Physical functioning (+, p=0.75), Physical limitations +, p=0.58), Emotional limitations (+, p= 0.58), Bodily pain (+, p=0.001), Energy (+, p= 0.01), Mental health (+, p=0.24), General health (+, p=0.006), Basic ADL (+, p<0.001), IADL (NS, p=0.65), Physical performance (+, p<0.0001) |
| Saltvedt, I., et al. (2002) [8], Norway *** | RCT, assessment at hospital admission, follow-up 3, 6 and 12 months, and discharge. All patients (frail elderly) $\geq$ 75 years, admitted to a GEM unit or a general unit at a university hospital, with a frail condition including chronic disability, acute impairment of single ADL, mild/moderate dementia, confusion, depression, imbalance/dizziness, falls, impaired mobility, urinary incontinence, malnutrition, polypharmacy, vision/hearing impairment, social problems, or prolonged bedrest. Patients were excluded if they had cancer with metastasis, other diseases with survival rate of 6 months, and severe dementia before admission. | n=127, mean age=81.8$\pm$4.8, male=36%, female=64%, 73% living alone. Previous diagnosis: 36% heart disease, 24% infectious disease, 21% gastrointestinal disorder, 19% cerebrovascular disease, 16% endocrine disease, 14% airway disease, 12% cancer, 32% other. Causes of death 12 months after inclusion: 40% heart disease, 34% infectious disease, 3% cerebrovascular disease, 9% cancer, 14% other. | n=127, mean age=82.4$\pm$5.2, male=34%, female=66%, 67% living alone. Previous diagnosis: 46% heart disease, 17% infectious disease, 17% gastrointestinal disorder, 13% cerebrovascular disease, 13% endocrine disease, 7% airway disease, 9% cancer, 35% other. Causes of death 12 months after inclusion: 54% heart disease, 14% infectious disease, 12% cerebrovascular disease, 14% cancer, 7% other. | A GEM unit as part of the hospital wards with a core interdisciplinary team of nurses with formal training in geriatric nursing, one physician per 5 beds, two occupational therapists, and physiotherapist. Designed with interdisciplinary assessment, comprehensive disorder evaluation, patient-centred care for prevention of iatrogenic conditions and complications, early mobilization and rehabilitation, early discharge planning aligned with home services, home visit follow-up (if it required). | Patients assigned to usual care or the control group received treatment as usual with the same number of nurses per bed as GEM unit, one physician per 5–10 beds, part-time (50%) occupational therapist, and 5 physiotherapists. | Mortality 3 months (+, p=0.04), Mortality 6 months (+, p=0.02), Mortality 12 months (+, p=0.06), LOS before inclusion (+, p=0.09), LOS after inclusion (-, P<0.001) |
| Saltvedt, I., et al. (2004) [9], Norway *** | RCT, assessment at hospital admission, follow-up 6 months, and discharge. All patients (frail elderly) $\geq$ 75 years, admitted to a GEM unit or a general unit at a university hospital, with a frail condition including chronic disability, acute impairment of single ADL, mild/moderate dementia, confusion, depression, imbalance/dizziness, falls, impaired mobility, urinary incontinence, malnutrition, polypharmacy, vision/hearing impairment, social problems, or prolonged bedrest. Patients were excluded if they had cancer with metastasis, other diseases. | n=127, mean age=81.8$\pm$4.8, male=36%, female=64%, 73% living alone. Previous diagnosis: 36% heart disease, 24% infectious disease, 21% gastrointestinal disorder, 19% cerebrovascular disease, 16% endocrine disease, 14% airway disease, 12% cancer, 32% other. Diagnosis at discharge: 57% heart disease, 16% infectious disease, 17% gastrointestinal disease, 30% cerebrovascular disease, 19% endocrine disease, 13% airway disease, 9% cancer, 38% psychiatric disorder, 40% other. | n=127, mean age=82.4$\pm$5.2, male=34%, female=66%, 67% living alone. Previous diagnosis: 46% heart disease, 17% infectious disease, 17% gastrointestinal disorder, 13% cerebrovascular disease, 13% endocrine disease, 7% airway disease, 9% cancer, 35% other. Diagnosis at discharge: 54% heart disease, 20% infectious disease, 12% gastrointestinal disease, 22% cerebrovascular disease, 17% endocrine disease, 9% airway disease, 8% cancer, 7% psychiatric disorder, 54% other. | A GEM unit as part of the hospital wards with a core interdisciplinary team of nurses with formal training in geriatric nursing, one geriatrician, one physician per 5 beds, two occupational therapists, and physiotherapist. Designed with interdisciplinary assessment, comprehensive disorder evaluation, patient-centred care for prevention of iatrogenic conditions and complications, early mobilization and rehabilitation, early discharge planning aligned with home services, home visit follow-up (if it required). | Patients assigned to usual care or the control group received treatment as usual with the same number of nurses as GEM unit, one physician, part-time occupational therapist, and 5 physiotherapists. Physiotherapy and occupational therapy were given when prescribed by the doctor. | Mortality 3 months (+, p=0.04), Mortality 6 months (+,p=0.02), Mortality 12 months (+, p=0.06), Readmission (NS, no p-val), LOS (-, p <0.001), Admission/Institutionalisation (NS, no p-val) |
| Saltvedt, I., et al. (2005) [10], Norway *** | RCT, assessment at hospital admission, follow-up 3, 6 and 12 months, and discharge. All patients (frail elderly) $\geq$ 75 years, admitted to a GEM unit or a general unit as emergencies at a university hospital, with a frail condition including chronic disability, acute impairment of single ADL, mild/moderate dementia, confusion, depression, imbalance/dizziness, falls, impaired mobility, urinary incontinence, malnutrition, polypharmacy, vision/hearing impairment, social problems, or prolonged bedrest. Patients were excluded if they had cancer with metastasis, severe dementia, if they lived in a nursing home, or fully recovered. | n=127, mean age=81.8$\pm$4.8, male=36%, female=64%, 73% living alone, 32% using $\geq$ 5 drugs continuously at inclusion, 29% using $\geq$ 5 drugs continuously at discharge. Drug profile (with effect on diagnostic categories) at inclusion (I) and discharge (D): I: 45%,D: 48% gastrointestinal system and diabetes, I: 54%, D: 60% blood and blood-building organs, I: 63%, D: 58% cardiovascular system, I: 6%, D: 18% urogenital system, I: 13%, D: 17% endocrine system, I: 25%, D: 13% systemic infections, I: 6%, D: 6% musculoskeletal system, I: 31%, D: 35% central nervous system, I: 17%, D: 9% respiratory system. | n=127, mean age=82.4$\pm$5.2, male=34%, female=66%, 67% living alone, 39% using $\geq$ 5 drugs continuously at inclusion, 39% using $\geq$ 5 drugs continuously at discharge. Drug profile (with effect on diagnostic categories) at inclusion (I) and discharge (D): I: 51%,D: 52% gastrointestinal system and diabetes, I: 54%, D: 54% blood and blood-building organs, I: 77%, D: 76% cardiovascular system, I: 6%, D: 4% urogenital system, I: 12%, D: 16% endocrine system, I: 31%, D: 18% systemic infections, I: 13%, D: 13% musculoskeletal system, I: 25%, D: 31% central nervous system, I: 17%, D: 10% respiratory system. | A GEM unit as part of the hospital wards with a core interdisciplinary team of nurses, geriatricians, residents, enrolled nurses, occupational therapists, physiotherapists. Designed with interdisciplinary and comprehensive geriatric assessment, comprehensive disorder evaluation, patient-centred care for prevention of iatrogenic conditions and complications, early mobilization and rehabilitation, early discharge planning aligned with home services, home visit follow-up (if it required). No clinical pharmacologist/pharmacist was employed/used as a consultant in either of the groups. | Patients assigned to usual care or the control group received treatment as usual with the same number of nurses as GEM unit, including doctors and nurses, and enrolled nurses. Physiotherapy and occupational therapy were given if it was prescribed and required by the doctor. | Polypharmacy (NS), Scheduled drugs withdrawn (+, p=0.005). Drugs with anticholinergic effects (withdrawn) (+, p=0.003), cardiovascular drugs (withdrawn) (+, p<0.001), particularly digitalis glycosides (withdrawn) (+ p<0.001), and antipsychotic drugs (withdrawn) (+, p=0.009), LOS (-, p <0.001), Admission/Institutionalisation (NS, no p-val) |
| Coleman, E. A., et al. (2006) [11], USA | RCT, assessment at hospital admission, follow-up 1, 3 and 6 months, and discharge. All patients ≥ 65 years, with complex care needs requiring care across different health setting at a university hospital, with any of the following conditions: stroke, congestive heart failure, coronary artery disease, cardiac arrhythmias, chronic obstructive pulmonary disease, diabetes mellitus, spinal stenosis, hip fracture, peripheral vascular disease, deep venous thrombosis, and pulmonary embolism. Patients were excluded if they were not admitted to the participating delivery system’s contract hospital during the study period, from a long-term care facility, reside outside a predefined geographic area, not English speaking, if they have dementia, participating in another research. | n=379, mean age=76.6$\pm$7.1, male=51.7%, female=48.3, 31% living alone. Selected hospital discharge diagnosis: 2.4% stroke, 16.5% congestive heart failure, 14.1% coronary artery disease, 12.8%, cardiac arrhythmia, 16.8% chronic obstructive pulmonary disease, 2.7% diabetes mellitus, 4% hip fracture, 4.5% dehydration, 8% pneumonia. | n=371, mean age=76.4$\pm$6.8, male=47.7%, female=52.3%, 30.8% living alone. Selected hospital discharge diagnosis: 4.7% stroke, 13% congestive heart failure, 13.5% coronary artery disease, 19% cardiac arrhythmia, 18.5% chronic obstructive pulmonary disease, 2.8% diabetes mellitus, 3.6% hip fracture, 3.6% dehydration, 8.8% pneumonia. | A care transition intervention for older patients based on 4 pillars/domains, that were consequent of patient and caregiver feedback on most valuable attributes of care transitions. 4 pillars are (a) assistance with medication self-management, (b) a patient-centred record owned/maintained by the patient to simplify cross-site information transfer, (c) timely follow-up with primary or specialty care, (d) a list of “red flags” or risk factors indicative of a worsening condition including instructions on respond plan. These 4 pillars work based on two mechanisms designed to inspire patients and their caregivers to foster care management and permanency across settings: (I) a personal health record and (II) a series of visits/telephone calls with a transition coach. | Patients assigned to usual care or the control group were eligible to receive all standard and usual treatments and services ordered and provided by their primary physicians and nurses. | Readmission at 30 days (+, p=0.048) and at 90 days (+, p=0.04), Costs (+, p=0.049), Mortality (NS, p=0.54), LOS (-, no p-val) |
| Phibbs, C. S., et al. (2006) [12], USA ** | RCT, assessment at hospital admission, follow-up 1 year and discharge. All patients (frail elderly) $\geq$ 70 years, hospitalized on a medical or surgical ward at a veteran’s university hospital, with an expected LOS of at least 2 days, and a frail-condition. The primary focus of this study is on resource use and costs. | Post-randomization costs including index hospitalization: GEMU/UCOP = 36,592$\pm$1844, GEMU/GEMC = 35,935$\pm$1829, post-randomization cost of index hospitalization: GEMU/UCOP = 13,086$\pm$951, GEMU/GEMC = 13,815$\pm$1516, total cost after index discharge: GEMU/UCOP = 23,506$\pm$1540, GEMU/GEMC = 22,121$\pm$798 | Post-randomization costs including index hospitalization: UCIP/UCOP = 38,624$\pm$2037, UCIP/GEMC = 35,951$\pm$1827, post-randomization cost of index hospitalization: UCIP/UCOP = 10,823$\pm$1821 , UCIP/GEMC = 10,693$\pm$846, total cost after index discharge: UCIP/UCOP = 27,801$\pm$831 , UCIP/GEMC = 25,258$\pm$1566 | A GEM unit as part of the hospital inpatient and outpatient units. Designed with interdisciplinary and comprehensive geriatric assessment, comprehensive disorder evaluation, patient-centred care for prevention of iatrogenic conditions and complications, early mobilization and rehabilitation, early discharge planning aligned with home services, home visit follow-up (if its required). | Patients assigned to usual care or the control group were eligible to receive all standard and usual treatments and services ordered and provided by their primary physicians and nurses. | Nursing home placement (+, p= 0.001), Resource used post discharge (+, p=0.001), Mortality (NS, no p-val), Cost (+, p= 0.29), LOS (-, no p-val) |
| Saltvedt, I., et al. (2006) [13], Norway *** | RCT, assessment at hospital admission, follow-up 3, 6 and 12 months, and discharge. All patients (frail elderly) $\geq$ 75 years, admitted to a GEM unit or a general unit as emergencies at a university hospital, with a frail condition including chronic disability, acute impairment of single ADL, mild/moderate dementia, confusion, depression, imbalance/dizziness, falls, impaired mobility, urinary incontinence, malnutrition, polypharmacy, vision/hearing impairment, social problems, or prolonged bedrest. Patients were excluded if they had cancer with metastasis, severe dementia, if they lived in a nursing home, or fully recovered. | n=127, mean age=81.8$\pm$4.8, male=36%, female=64%. Previous diagnosis: 36% heart disease, 24% infectious disease, 21% gastrointestinal disorder, 19% cerebrovascular disease, 16% endocrine disease, 14% airway disease, 12% cancer, 32% other. Targeting criteria: 87% acute impairment of single ADL, 87% imbalance, dizziness, 43% impaired mobility, 41% chronic disability, 24% weight loss, 24% falls, 19% confusion, 17% vision/hearing impairment, 17% depression, 12% malnutrition, 11% moderate dementia, 9% urinary incontinence, 8% social problems, 4% polypharmacy, 2% prolonged bed rest | n=127, mean age=82.4$\pm$5.2, male= 34%, female=66%. Previous diagnosis: 46% heart disease, 17% infectious disease, 17% gastrointestinal disorder, 13% cerebrovascular disease, 13% endocrine disease, 7% airway disease, 9% cancer, 35% other. Targeting criteria: 86% acute impairment of single ADL, 85% imbalance, dizziness, 47% impaired mobility, 46% chronic disability, 16% weight loss, 25% falls, 24% confusion, 22% vision/hearing impairment, 19% depression, 10% malnutrition, 14% moderate dementia, 9% urinary incontinence, 6% social problems, 4% polypharmacy, 2% prolonged bed rest | A GEM unit as part of the hospital wards with a core interdisciplinary team of nurses, geriatricians, residents, enrolled nurses, occupational therapists, physiotherapists. Designed with interdisciplinary and comprehensive geriatric assessment, comprehensive disorder evaluation, patient-centred care for prevention of iatrogenic conditions and complications, early mobilization and rehabilitation, early discharge planning aligned with home services, home visit follow-up (if it required). No clinical pharmacologist/pharmacist was employed/used as a consultant in either of the groups. | Patients assigned to usual care or the control group received treatment as usual with the same number of nurses as GEM unit, including doctors and nurses, and enrolled nurses. Physiotherapy and occupational therapy were given if it was prescribed and required by the doctor. | Mortality 3 months (+, p=0.04), Mortality 6 months (+,p=0.02), Mortality 12 months (+, p=0.06), Readmission (NS, no p-val), LOS (-, p <0.001), Admission/Institutionalisation (NS, no p-val), Function, depression or general well-being (NS, no p-val), If the dead were included in the analysis for ADL dependency level at 3 months (+, p = 0.03). |
| Kircher, T. T., et al. (2007) [14], Germany | RCT, assessment at hospital admission, follow-up 3 and 12 months, and discharge. All patients (frail elderly) $\geq$ 65 years, functionally impaired patients, expected LOS $\geq$ 8 days, admitted to general medical or psychiatric units at five hospitals. Patients excluded if they were admitted from a nursing home, had previously hospitalised in a GEM unit, terminal condition (severe dementia), did not communicate in German, reside outside a predefined geographic area, would not need help at home or could not give informed consent. | n= 186, mean age=79.0$\pm$6.9, male=48%, female=52%, 39.3% had at least one hospitalisation in the past year, 5.3% used wheelchair, 52.7% vision impairment, 74% hearing impairment. | n=129, mean age=78.4$\pm$6.9, male=24%, female=86%, 29.4% had at least one hospitalisation in the past year, 3.9% used wheelchair, 58.9% vision impairment, 72.9% hearing impairment. | A GEM unit as part of the hospital wards with a core interdisciplinary team of nurse, geriatrician, resident, general practitioner, social worker, physiotherapist. Designed with interdisciplinary and comprehensive geriatric assessment, comprehensive disorder evaluation, patient-centred care for prevention complications, early mobilization and rehabilitation, early discharge planning aligned with home services, home visit and phone follow-up (if its required). | Patients assigned to usual care or the control group were eligible to receive all standard and usual treatments and services ordered and provided by their primary physicians and nurses. | Readmission/Rehospitalisation (12 months) (NS, p= 0.30), Nursing home placement (12 months) (NS, p=0.27), Mortality (12 months) (NS, p=0.56) |
| Tibaldi, V., et al. (2009) [15], Italy | RCT, assessment at hospital admission, follow-up 6 months, and discharge. All patients $\geq$ 75 years, with a pre-existing diagnosis of CHF with symptoms such as dyspnoea, fatigue caused by abnormal cardiac function, a persistent functional impairment, appropriate care supervision at home, living in the hospital at-home area, at least 1 previous admission for acute CHF, need for intravenous drug infusion, admitted to the ED at an urban university hospital. Patients were excluded if new-onset heart failure, absence of family/social support, need for mechanical ventilation, haemodialysis, intensive monitoring, severe dementia, severe renal impairment, hepatic failure, and planned cardiac surgery. | n=48, mean age=82.2$\pm$5.2, male=46%, female=54%. Symptoms on admission: 62% dyspnoea at rest, 29% dyspnoea at minimal effort, 48% orthopnoea, 19% paroxysmal nocturnal dyspnoea, 90% fatigue, 29% mental disorder or confusion. Clinical signs on admission: 44% pulmonary rales, 77% peripheral edema, 8% positive hepatojugular reflux, 23% jugular vein distention, 2% third heart sound, 21% tachycardia. Prevalent heart disease: 40% hypertensive cardiopathy, 27% ischemic cardiopathy or coronary artery disease, 17% heart valve disease, 10% dilative cardiomyopathy, 6% other. | n=53, mean age=80.1$\pm$4.9, male=57%, female=43%. Symptoms on admission: 72% dyspnoea at rest, 38% dyspnoea at minimal effort, 32% orthopnoea, 17% paroxysmal nocturnal dyspnoea, 55% fatigue, 23% mental disorder or confusion. Clinical signs on admission: 57% pulmonary rales, 77% peripheral edema, 13% positive hepatojugular reflux, 24% jugular vein distention, 8% third heart sound, 21% tachycardia. Prevalent heart disease: 34% hypertensive cardiopathy, 26% ischemic cardiopathy or coronary artery disease, 15% heart valve disease, 11% dilative cardiomyopathy, 9% other. | A GHHS unit as part of the hospital wards with a core interdisciplinary team of geriatrician, nurse, physiotherapist, social worker, and counsellor. Designed with frequent team medical review, early rehabilitation, home visit and follow-up, early discharge and referral planning, protocols for prevention of nosocomial infections, bed sores, and immobilization are routinely adopted for frail elderly inpatients. Also, as mentioned by the study from an administrative/legal, and financial standpoint, GHHS patients were considered hospital inpatients until discharge. | Patients assigned to the inpatient control group were eligible to receive all routine hospital care ordered and provided by their primary physicians and nurses. Protocols for prevention of nosocomial infections, bed sores, and immobilization are routinely adopted for frail elderly inpatients. | Mortality (6 months) (NS, p=0.83), Morbidity (infections, delirium, bed sores, deep vein thrombosis, and falls) during hospitalization: (+, p-val = 0.12-0.95), Admissions to a nursing home, and subsequent hospital admissions related to any cause: (+), LOS (-, p=0.001), Costs (+, p<0.001), IADL (NS, p=0.29) |
| Legrain, S., et al. (2011) [16], France | RCT, assessment at hospital admission, follow-up 3 and 6 months, and discharge. All patients $\geq$ 70 years admitted to the ED of 6 university hospitals. Patients were excluded if expected LOS $\leq$ 5 days, poor chance of survival (3 months), palliative care, previous participation in the OMAGE study or in another therapeutic trial, not French speaking, impossible to follow up, and absence of any health insurance. | n=317, mean age=85.5$\pm$6.0, male=30.3%, female=69.7%, 47.0% living alone, 33.7% had <5 ADLs 15 days before admission. Previous diagnosis: 22.1% dementia, 15.8% stroke, 67.2% hypertension, 17% diabetes mellitus, 16.1% heart failure, 29.3% coronary artery disease, 26.8% arrythmia, 10.1% chronic pulmonary insufficiency, 11% cancer. | n=348, mean age=86.4$\pm$6.3, male=37.4%, female=62.6%, 47.1% living alone, 36.0% had <5 ADLs 15 days before admission. Previous diagnosis: 22.1% dementia, 12.6% stroke, 65.5% hypertension, 14.7% diabetes mellitus, 14.9% heart failure, 28.5% coronary artery disease, 22.7% arrythmia, 12.9% chronic pulmonary insufficiency, 14.9% cancer. | An AGU unit as part of the hospital wards utilising OMAGE intervention with a core interdisciplinary team of IDG, geriatrician, general practitioner, nurses. Designed with comprehensive discharge planning, patient-centred care, comprehensive chronic treatment review, education on self-management of disease, enhanced transition-of-care communication, for patients with multiple chronic conditions. | Patients assigned to usual care or the control group were eligible to receive all standard and usual treatments and services from AGU team including CGA, without any involvement of the IDG. | Emergency hospitalization (+, p=0.03), NNT (6 months) (+, p=0.15), Readmission (3months) (+, p=0.01), Readmission (6months) (+, p=0.12), Mortality (3months) (+, p=0.63), Mortality (6months) (+, p=0.74) |
| Wald, H. L., et al. (2011) [17], USA | QRCT, assessment at hospital admission, and discharge. All patients (frail elderly) $\geq$ 70 years, admitted to hospitalist-ACE service or general internal medicine unit and not a speciality unit and subspeciality service such as cardiology, pulmonary, or oncology, and not transferred to intensive care or orthopaedic surgery units. | n=122, mean age=80.5$\pm$6.5, male=47.5%, female=52.5%. Primary diagnosis: 27.9% pulmonary, 15.6% general medicine, 13.9% surgery, 9.8% cardiology, 8.2% nephrology. | n=95, mean age=80.7$\pm$7.0, male=41%, female=59%. Primary diagnosis: 28.4% pulmonary, 11.6% general medicine, 11.6% surgery, 6.3% cardiology, 7.4% nephrology. | A hospitalist-ACE service as part of the hospital wards with a core interdisciplinary team of geriatricians, registered nurses, social workers, physiotherapists, and nutritionists. Designed with selected hospitalist attendings, daily interdisciplinary rounds, standardized geriatric assessment, clinical focus on mitigating harm and discharge planning, novel inpatient geriatrics set of courses. | Patients assigned to the control group were eligible to receive all standard and usual treatments and services ordered and provided by their primary physicians and nurses. | Functional status (+ p<0.0001), Cognitive status (+, p=0.02), Physical restraints (NS), Sleep aids (NS), falls (NS), discharge location (NS), LOS (NS, p=0.52), Cost (-, p=0.12), Readmission (NS, p=0.50) |
| Barnes, D. E., et al. (2012) [18], USA | RCT, assessment at hospital admission, and discharge. All patients (community dwelling adults) $\geq$ 70 years, admitted to general medical care, not admitted (not requiring upon admission) to a speciality unit including intensive care, cardiology, oncology, telemetry at a university hospital. | n=858, mean age=80.6$\pm$7.3, male=33%, female=67%. Reason for admission: 11% neurologic, 14% cardiovascular, 12% pneumonia/infection, 21% pulmonary, 2% hematologic, 20% gastrointestinal, 18% metabolic. Coexisting conditions: 28% CHF, 7% cancer, 17% CLD, 15% myocardial infarction, 17% cerebrovascular disease, 18% dementia, 5% peripheral vascular disease. | n=774, mean age=80.6$\pm$7.4, male=33%, female=67%. Reason for admission: 10% neurologic, 14% cardiovascular, 14% pneumonia/infection, 22% pulmonary, 3% hematologic, 20% gastrointestinal, 17% metabolic. Coexisting conditions: 26% CHF, 7% cancer, 17% CLD, 13% myocardial infarction, 16% cerebrovascular disease, 19% dementia, 8% peripheral vascular disease. | ACE unit specifically designed for the study with a core team of geriatrician, registered nurses, geriatric clinical nurse, social worker, physiotherapist, and nutritionist. Designed with frequent medical review, early discharge planning, prepared environment, patient-centred care, and early rehabilitation with an emphasis on independence and early rehabilitation. | Patients assigned to the control group were eligible to receive all standard and usual treatments and services ordered and provided by their primary physicians and nurses in the general inpatient unit for younger and older patients resided together. | LOS (+), Costs (+), ADL (NS), IADL (NS), Mobility (NS), Readmission (NS), Mortality (+, p=0.23), Discharged to home (NS), |
| Buurman, B. M., et al. (2016) [19], Netherlands | RCT, assessment at hospital admission, follow-up 1 and 6 months, discharge. All patients $\geq$ 65 years, were acutely admitted for at least 48 hours to an internal medicine department and were at risk for functional decline at 3 university hospitals. Functional decline defined using ISAR with a score of 2 or higher to be at increased risk. Also, geriatric conditions are considered as polypharmacy, incontinence, malnutrition, delirium, and fall risk. | n=337, mean age=79.7$\pm$7.3, male=42.1%, female=57.9%, 67.9% independent, 7.7% with assisted living. Admission diagnosis: 30.3% infection, 14.5% gastrointestinal, 9.2% cardiac, 11.3% respiratory, 2.7% cancer, 6.8% electrolyte disturbances, 3.3% renal, 22% Other. CGA at admission: 80.1% polypharmacy, 34.7% incontinence, 24.7% indwelling urinary catheter, 45.6% dizziness, 53.3% malnutrition, 52.1% pain, 40.4% cognitively impaired, 34.3% sad or depressive symptoms in the past month, 31.5% less interest in activities, 45.3% fall in the past 6-month, 58% sleep disturbances, 25.3% hearing impairment, 19.6% visual impairment. | n=337, mean age=80.0$\pm$7.8, male=42.1%, female= 57.9%, 67.1% independent, 9.3% with assisted living. Admission diagnosis: 27.9% infection, 13.9% gastrointestinal, 12.2% cardiac, 11.9% respiratory, 6.5% cancer, 4.7% electrolyte disturbances, 3.6% renal, 19.3% Other. CGA at admission: 78.8% polypharmacy, 35.5% incontinence, 24.4% indwelling urinary catheter, 48.8% dizziness, 51.1% malnutrition, 46.2% pain, 38% cognitively impaired, 37.9% sad or depressive symptoms in the past month, 39.2% less interest in activities, 52.4% fall in the past 6-month, 51.1% sleep disturbances, 24.3% hearing impairment, 25.1% visual impairment. | A CGA-based intervention as a transitional care bridge program with a core interdisciplinary team of registered nurse, CCRN, general practitioner, geriatrician, geriatric-trained registered nurse, physical therapist, dietician. Designed with CGA-based approach involving the provision of comprehensive care and treatment plan, active follow-up as a home visit, multidisciplinary team care, and multidisciplinary team support. | Patients assigned to the control group were eligible to receive all standard and usual treatments and services ordered and provided by their primary physicians and nurses. Also, CGA evaluation was also conducted for control group without the intervention. | ADL (NS), Mortality (6 months) (+, p=0.045), Cognitive functioning (NS), Readmission (NS), time to discharge from a nursing home (NS), institutionalization (NS) |
| Westgard, T., et al. (2020) [20], Sweden | RCT, assessment at hospital admission, follow-up 1, 6 and 12 months, and discharge. All patients (frail elderly) $\geq$ 75 years, required an unplanned acute medical admission and admitted to the emergency department at a university hospital, and were not admitted via a fast-track pathway including stroke, coronary infarct, hip fracture. Frailty indicators were used based on Fried’s criteria, with the addition of visual and cognitive impairment because of the high impact on disability. | n=78, mean age=87.5, male=39.7%, female=60.3%, 65.5% living alone, 5.1% independent ADL, 35.9% decreased level of ADL one to six months, 33.3% satisfied with ADL, 14.1% satisfied with physical health, 32% satisfied with mental health, 73% with more than 5 frailty indicator. | n=77, mean age=86.2, male= 44.20%, female =55.8%, 62.3% living alone, 6.5% independent ADL, 39% decreased level of ADL one to six months, 41.6% satisfied with ADL, 9% satisfied with physical health, 40.3% satisfied with mental health, 67.6% with more than 5 frailty indicator. | A CGA-based intervention as part of a geriatric acute medical ward for frail older patients with a core interdisciplinary team of geriatric trained medical doctor, nurse, nurse assistant, occupational therapist, and physical therapist, social worker, and nutritionist. Designed with CGA-based approach involving a patient-centred care of holistic and comprehensive evaluation of the patient’s medical status, self-assessed health, functional status, psychological status, social situation, and environment, nutritional support, social support. Active follow-up and multidisciplinary team support for long-term care needs was provided when it was required. | Patients assigned to the control group were eligible to receive all standard and usual treatments and services on the acute medical wards by a medical doctor, nurse, and nurse assistant. Also, the control group received usual services without a specialized multidisciplinary team practicing the CGA. | IIT (NS), Improved ADL (1 month) (+, p=0.249), ADL (6 month) (NS, p=0.611), ADL (1 to 6 months) (NS, p=0.452), Functional decline (NS). |
| ¶ Outcomes are primary and secondary outcomes as described in the eligible research studies of the systematic literature review.  § + indicates statistically in favour of the intervention group, and if p-value was not given it is p<0.05; -, statistically in favour of the control group and if p-value was not given it is p<0.05; p-val, P-Value; NS, not significant (or if no p-values were given by the study). Unknown/unclear studies with no statistical analysis were excluded from this table.  */**/***/…: Same group of studies (same RCT) – Group */Group**/Group***/…  RCT indicates randomised controlled trial; MRCT, multisite randomized control trial; QRCT, quasi-randomized control trial; ACE, acute care for elderly; AGU, acute geriatric units; UCIP, usual-care inpatient; UCOP, usual-care outpatient; GEMU, geriatric evaluation and management unit; GEMC, geriatric evaluation and management clinic; GHHS, geriatric hospital-at-home service; GRACE, geriatric resources for assessment and care of elders; RAC, residential aged care; DHB, district health board; ARCHUS, aged residential care healthcare utilization study; ED, emergency department; GEM, geriatric evaluation and management; OMAGE, Optimization of Medication in AGEd; AGe-FIT, ambulatory geriatric assessment - frailty intervention trial; NNT, number needed to treat; IDG, intervention-dedicated geriatrician; LOS, length of stay; CLD, chronic lung disease; CHF, chronic heart failure; ADL, activity of daily living; IADL, instrumental activity of daily living; ISAR, identification of seniors at risk; CGA, comprehensive geriatric assessment; SPMSQ, short portable mental status questionnaire; GNS, gerontology nurse specialist; HRQoL, health-related quality of life; CCRN, community care registered nurse; IIT, intention to treat. | | | | | | |

**Appendix S2–** Meta-Analysis Plots & Outputs

**Improved ADL**

**
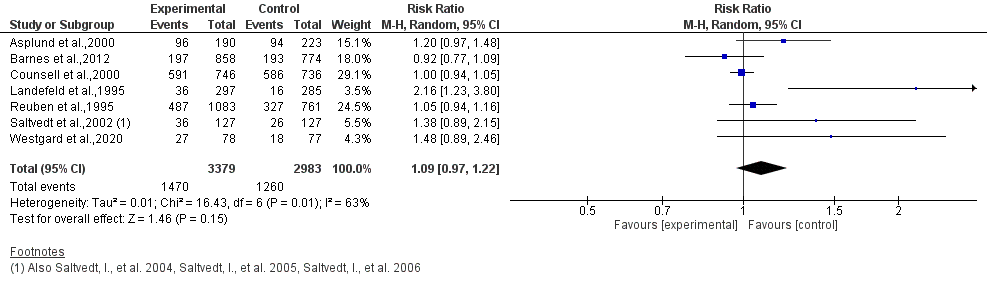
**

**Improved ADL [Outlier Removed]**

**
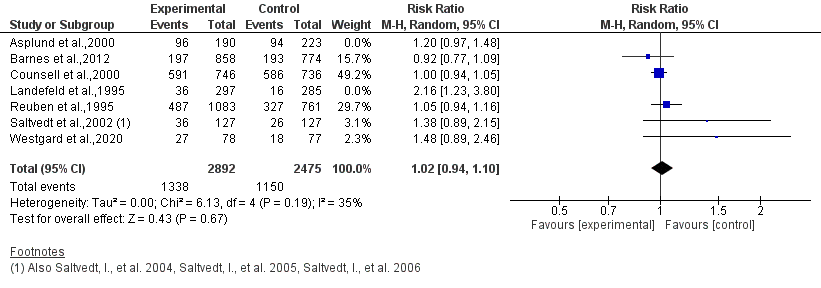
**

**Hospital Readmission**

**
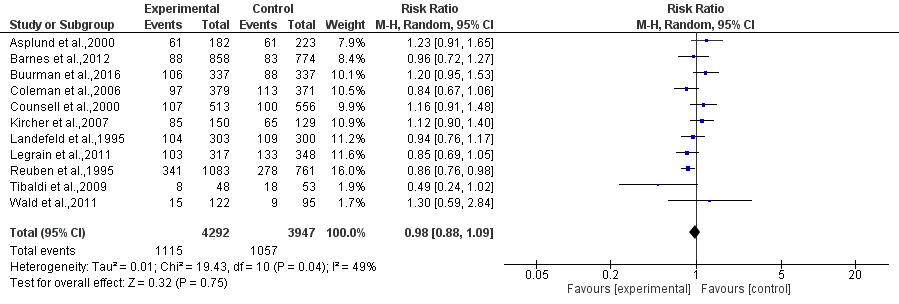
**

**Hospital Readmission [Outlier Removed]**

**
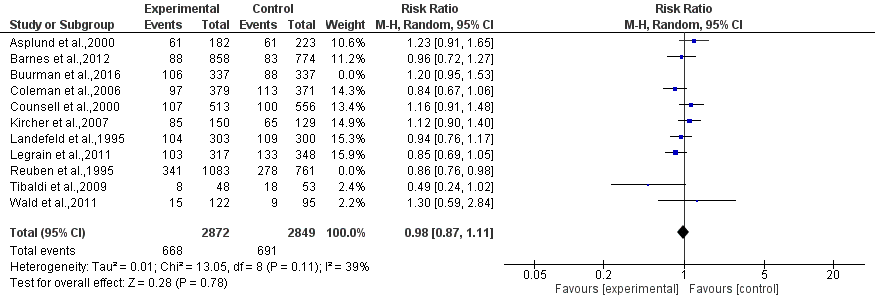
**

**Hospital Costs (US $)**

**
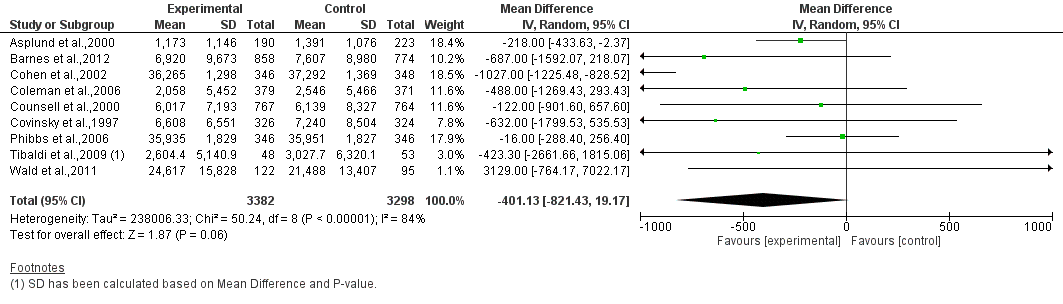
**

**Hospital Costs (US $) [Outlier Removed]**

**
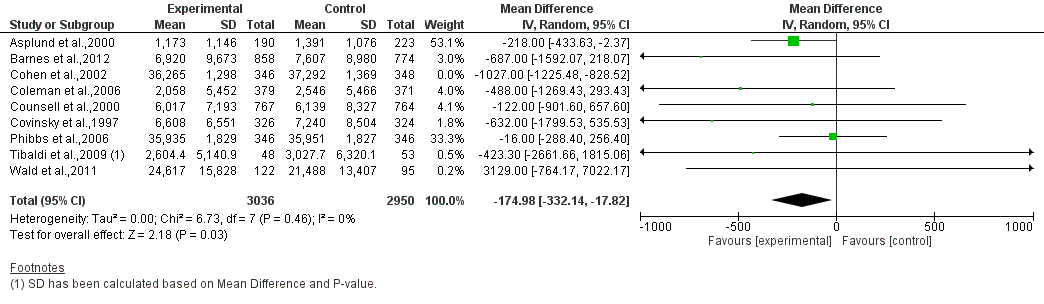
**

**Mortality**

**
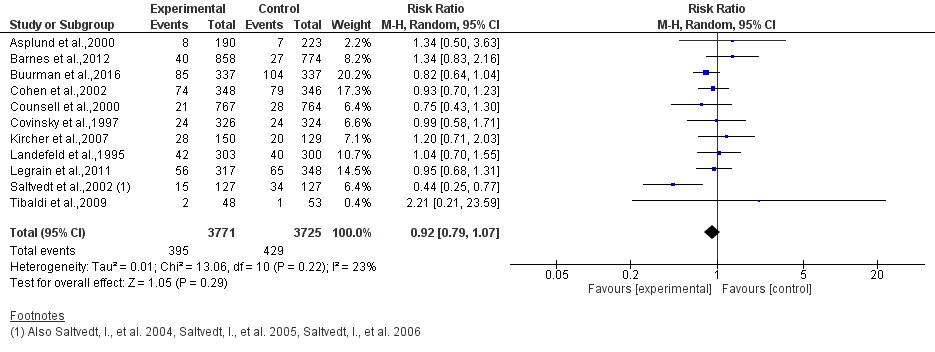
**

**Case Fatality at Discharge**

**
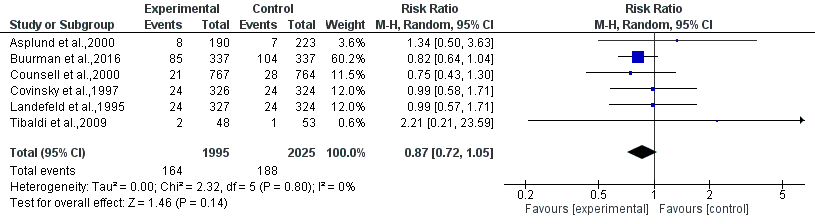
**

**Case Fatality at Follow-up
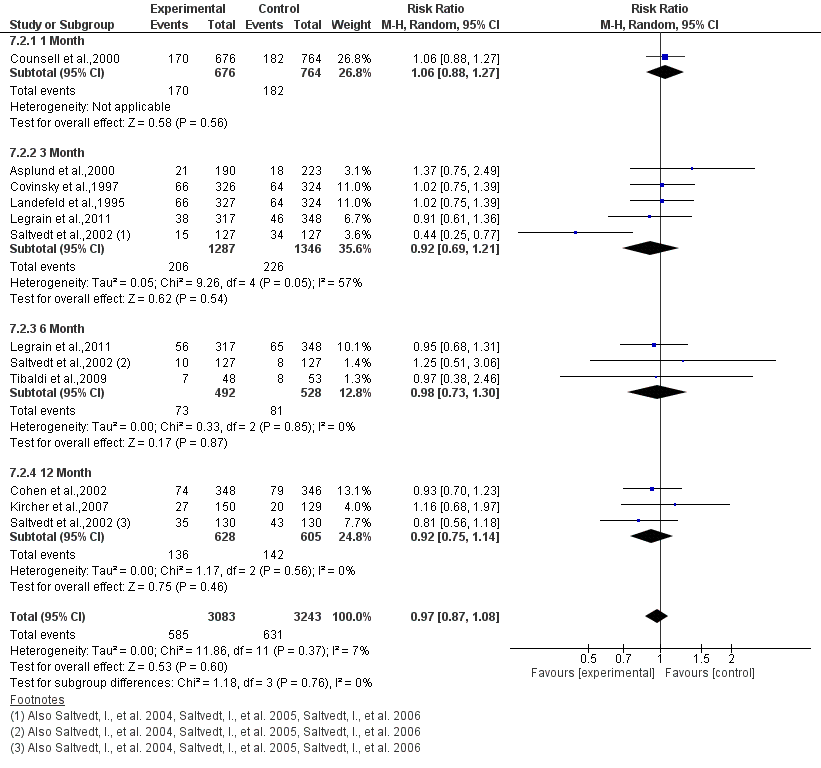
**

**Functional Outcome/Decline**

**
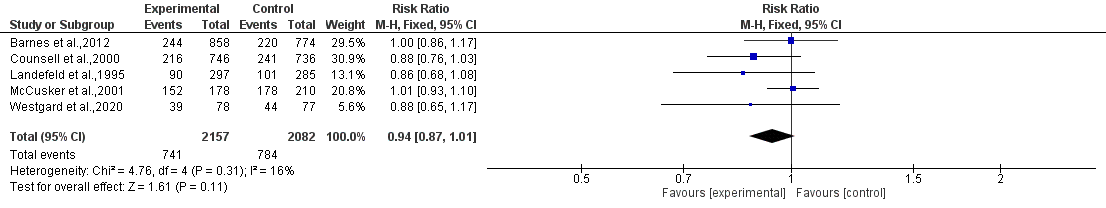
**

**LOS (Days)**

**
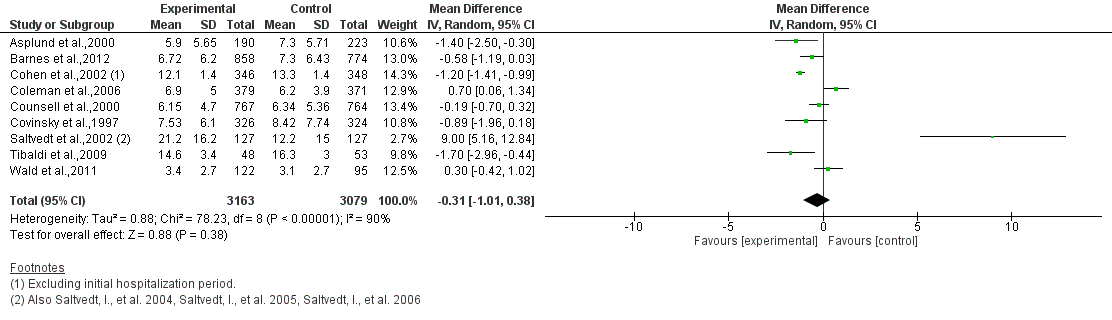
**

**LOS (Days) [Outlier Removed]**

**
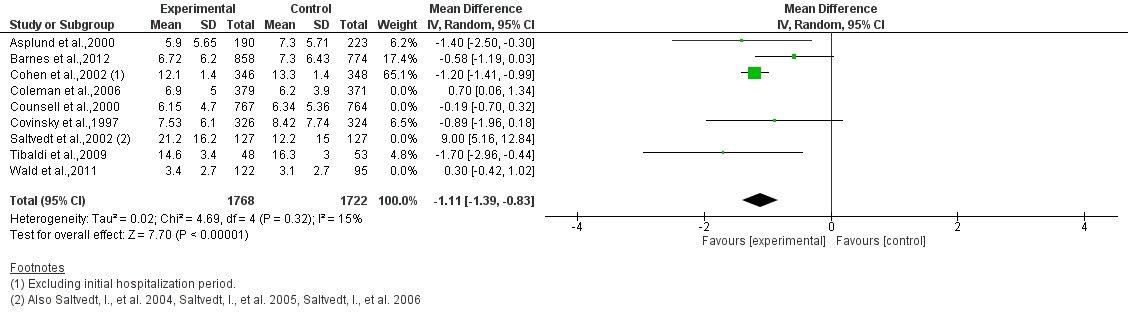
**

**Living Situation at Discharge**

**
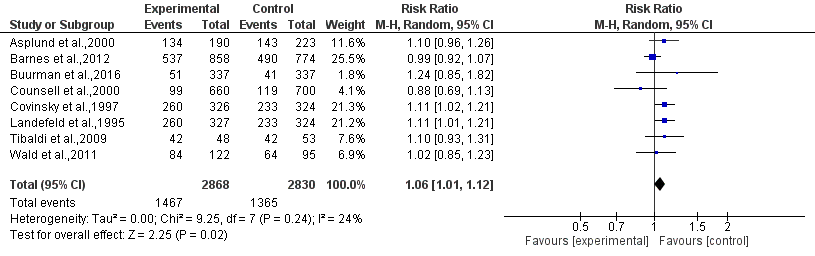
**

**Living Situation at Follow-up**

**
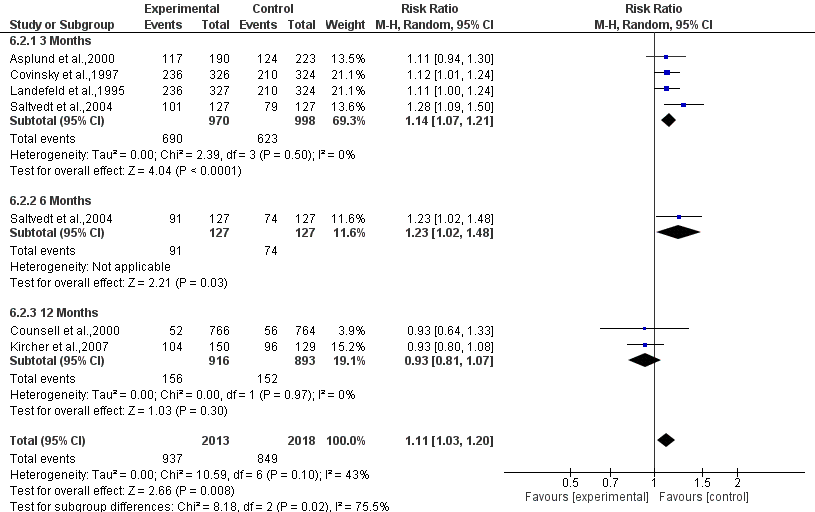
**

**Supplimentary Material References**

1. Landefeld, C.S., et al., *A Randomized Trial of Care in a Hospital Medical Unit Especially Designed to Improve the Functional Outcomes of Acutely Ill Older Patients.* New England Journal of Medicine, 1995. **332**(20): p. 1338-1344.

2. Reuben, D.B., et al., *A randomized trial of comprehensive geriatric assessment in the care of hospitalized patients.* N Engl J Med, 1995. **332**(20): p. 1345-50.

3. Covinsky, K.E., et al., *Do Acute Care for Elders Units Increase Hospital Costs? A Cost Analysis Using the Hospital Perspective.* Journal of the American Geriatrics Society, 1997. **45**(6): p. 729-734.

4. Counsell, S.R., et al., *Effects of a multicomponent intervention on functional outcomes and process of care in hospitalized older patients: a randomized controlled trial of Acute Care for Elders (ACE) in a community hospital.* J Am Geriatr Soc, 2000. **48**(12): p. 1572-81.

5. Asplund, K., et al., *Geriatric-based versus general wards for older acute medical patients: A randomized comparison of outcomes and use of resources.* Journal of the American Geriatrics Society, 2000. **48**(11): p. 1381-1388.

6. McCusker, J., et al., *Rapid emergency department intervention for older people reduces risk of functional decline: results of a multicenter randomized trial.* J Am Geriatr Soc, 2001. **49**(10): p. 1272-81.

7. Cohen, H.J., et al., *A controlled trial of inpatient and outpatient geriatric evaluation and management.* New England Journal of Medicine, 2002. **346**(12): p. 905-912.

8. Saltvedt, I., et al., *Reduced mortality in treating acutely sick, frail older patients in a geriatric evaluation and management unit. A prospective randomized trial.* J Am Geriatr Soc, 2002. **50**(5): p. 792-8.

9. Saltvedt, I., et al., *Acute geriatric intervention increases the number of patients able to live at home. A prospective randomized study.* Aging Clinical and Experimental Research, 2004. **16**(4): p. 300-306.

10. Saltvedt, I., et al., *Patterns of drug prescription in a geriatric evaluation and management unit as compared with the general medical wards: a randomised study.* Eur J Clin Pharmacol, 2005. **61**(12): p. 921-8.

11. Coleman, E.A., et al., *The care transitions intervention: results of a randomized controlled trial.* Arch Intern Med, 2006. **166**(17): p. 1822-8.

12. Phibbs, C.S., et al., *The effect of geriatrics evaluation and management on nursing home use and health care costs - Results from a randomized trial.* Medical Care, 2006. **44**(1): p. 91-95.

13. Saltvedt, I., et al., *Randomised trial of in-hospital geriatric intervention: impact on function and morale.* Gerontology, 2006. **52**(4): p. 223-30.

14. Kircher, T.T., et al., *A randomised trial of a geriatric evaluation and management consultation services in frail hospitalised patients.* Age Ageing, 2007. **36**(1): p. 36-42.

15. Tibaldi, V., et al., *Hospital at Home for Elderly Patients With Acute Decompensation of Chronic Heart Failure A Prospective Randomized Controlled Trial.* Archives of Internal Medicine, 2009. **169**(17): p. 1569-1575.

16. Legrain, S., et al., *A new multimodal geriatric discharge-planning intervention to prevent emergency visits and rehospitalizations of older adults: the optimization of medication in AGEd multicenter randomized controlled trial.* J Am Geriatr Soc, 2011. **59**(11): p. 2017-28.

17. Wald, H.L., et al., *Evaluation of a hospitalist-run acute care for the elderly service.* J Hosp Med, 2011. **6**(6): p. 313-21.

18. Barnes, D.E., et al., *Acute care for elders units produced shorter hospital stays at lower cost while maintaining patients' functional status.* Health Aff (Millwood), 2012. **31**(6): p. 1227-36.

19. Buurman, B.M., et al., *Comprehensive Geriatric Assessment and Transitional Care in Acutely Hospitalized Patients: The Transitional Care Bridge Randomized Clinical Trial.* JAMA Intern Med, 2016. **176**(3): p. 302-9.

20. Boockvar, K.S., et al., *Hospital Elder Life Program in Long-Term Care (HELP-LTC): A Cluster Randomized Controlled Trial.* J Am Geriatr Soc, 2020.

21. Jadad, A.R., et al., *Assessing the quality of reports of randomized clinical trials: Is blinding necessary?* Controlled Clinical Trials, 1996. **17**(1): p. 1-12.

1. Keywords for text word search in titles/abstracts using other research databases were extracted from these MeSH keyboards and subject headings. [↑](#footnote-ref-1)
